# Proteomic Analysis of *Neisseria gonorrhoeae* Biofilms Shows Shift to Anaerobic Respiration and Changes in Nutrient Transport and Outermembrane Proteins

**DOI:** 10.1371/journal.pone.0038303

**Published:** 2012-06-06

**Authors:** Nancy J. Phillips, Christopher T. Steichen, Birgit Schilling, Deborah M. B. Post, Richard K. Niles, Thomas B. Bair, Megan L. Falsetta, Michael A. Apicella, Bradford W. Gibson

**Affiliations:** 1 Department of Pharmaceutical Chemistry, University of California San Francisco, San Francisco, California, United States of America; 2 Department of Microbiology, College of Medicine, The University of Iowa, Iowa City, Iowa, United States of America; 3 Buck Institute for Research on Aging, Novato, California, United States of America; 4 Department of Obstetrics, Gynecology, and Reproductive Biology, University of California San Francisco, San Francisco, California, United States of America; University of Minho, Portugal

## Abstract

*Neisseria gonorrhoeae*, the causative agent of gonorrhea, can form biofilms *in vitro* and *in vivo*. In biofilms, the organism is more resistant to antibiotic treatment and can serve as a reservoir for chronic infection. We have used stable isotope labeling by amino acids in cell culture (SILAC) to compare protein expression in biofilm and planktonic organisms. Two parallel populations of *N. gonorrhoeae* strain 1291, which is an arginine auxotroph, were grown for 48 h in continuous-flow chambers over glass, one supplemented with ^13^C_6_-arginine for planktonic organisms and the other with unlabeled arginine for biofilm growth. The biofilm and planktonic cells were harvested and lysed separately, and fractionated into three sequential protein extracts. Corresponding heavy (H) planktonic and light (L) biofilm protein extracts were mixed and separated by 1D SDS-PAGE gels, and samples were extensively analyzed by liquid chromatography-mass spectrometry. Overall, 757 proteins were identified, and 152 unique proteins met a 1.5-fold cutoff threshold for differential expression with p-values <0.05. Comparing biofilm to planktonic organisms, this set included 73 upregulated and 54 downregulated proteins. Nearly a third of the upregulated proteins were involved in energy metabolism, with cell envelope proteins making up the next largest group. Of the downregulated proteins, the largest groups were involved in protein synthesis and energy metabolism. These proteomics results were compared with our previously reported results from transcriptional profiling of gonococcal biofilms using microarrays. Nitrite reductase and cytochrome c peroxidase, key enzymes required for anaerobic growth, were detected as highly upregulated in both the proteomic and transcriptomic datasets. These and other protein expression changes observed in the present study were consistent with a shift to anaerobic respiration in gonococcal biofilms, although changes in membrane proteins not explicitly related to this shift may have other functions.

## Introduction

*Neisseria gonorrhoeae*, a Gram-negative mucosal pathogen, is the causative agent of the sexually transmitted disease gonorrhea. It is a disease of global importance for which there is not yet an effective vaccine. Treatment of gonococcal infections usually involves single-dose antibiotic therapy. However, in recent years, the gonococcus has become progressively resistant to a wide range of antibiotics [1], [2], [3]. Recent studies showing gonococcal resistance to extended spectrum cephalosporins have suggested that the single dose therapy used since the 1940’s may no longer be a reasonable treatment option [1], [3]. Additionally, infections in women often go undetected, and thus untreated, which can lead to serious upper genital tract infections and pelvic inflammatory disease [4].

Recently, it was shown that *N. gonorrhoeae* can form biofilms on abiotic surfaces and over primary urethral and cervical epithelial cells [5]. Biopsy evidence has also indicated that biofilms are present during natural cervical infections [6]. In the biofilm growth form, bacteria exhibit increased resistance to clearance by host defenses and antibiotic treatment [7], [8], [9]. Consequently, naturally occurring biofilms may be a factor leading both to persistent *N. gonorrhoeae* infections in women and antibiotic resistance.

Biofilms are structured communities of bacteria that exist within a self-produced extracellular matrix [10]. Our recent studies have shown that a principal component of the matrix of the gonococcal biofilm is DNA produced by the organism [11], although membranous networks can also be observed throughout the biofilm that are presumed to arise from blebbing of the outer membrane [5], [6]. These networks appear to be related to gonococcal blebbing as piliated gonococcal msbB mutants that are defective in membrane blebbing fail to form biofilms [11]. In addition, blebs have been shown to act as a vehicle for the extracellular transport of gonococcal DNA [12]. Our studies would suggest this factor combined with death of organisms in the population contribute to the DNA within the biofilm matrix. Organisms living in biofilms differ physiologically from bacteria growing in a free-swimming planktonic state. However, it is now recognized that within bacterial biofilms there is also a great deal of physiological heterogeneity [13]. There are concentration gradients of oxygen, nutrients, waste products and secreted bacterial signaling compounds that alter the microenvironment at different spatial locations within a biofilm. Thus, cells within the interior of a mature biofilm experience very different conditions than bacteria at the bulk-fluid interface. Additionally, planktonic cells are continually released from the upper surface of a mature biofilm, further highlighting the diversified nature of such bacterial communities.

Given the importance of biofilm populations to disease, it is critical to better understand the biochemical signals that regulate biofilm formation and maintenance. During the past decade, there have been various genomic [14], [15] and proteomic [16] studies of biofilm formation, driven in part by technical advances in microarrays and mass spectrometry-based proteomic technologies. A central question in these studies is how planktonic bacteria alter their gene and protein expression patterns to adapt to biofilm colony formation. In the most heavily studied biofilm organism, *Pseudomonas aeruginosa* [17], [18], [19], [20], as well as in other organisms such as *Escherichia coli* [21], [22], [23], [24], *Staphylococcus aureus* [25], [26], and *Neisseria meningitidis* [27], [28], transcriptomic or proteomic studies have suggested that the shift to biofilm growth entails adaptations to oxygen and nutrient limitations, higher cell density, and stressful conditions. In general, the biofilm phenotype appears to involve differential expression of genes and proteins that planktonic cells can also express under specific environmental conditions. There is also some indication that overall gene expression in bacterial biofilms is more closely related to that of stationary phase planktonic cells [29]. While some overarching themes have emerged from these studies, it is also becoming clear that patterns of gene and protein expression in bacterial biofilms are organism-specific and are likely influenced by the environmental niche occupied by the organism.

We recently compared the transcriptional profiles of biofilm and planktonic populations of *N. gonorrhoeae* in an effort to identify biosynthetic pathways important for the development of gonococcal biofilms [30]. In this comparison, 3.8% of the *N. gonorrhoeae* genome was found to be differentially regulated. Three of the key genes that were upregulated in the biofilm organisms were all required for anaerobic respiration: nitrite reductase (*aniA*), nitric oxide reductase (*norB*), and cytochrome c peroxidase (*ccp*). These observations support the notion that gonococcal biofilms grow anaerobically or microaerobically. Many of the genes downregulated in the biofilm organisms belonged to the *nuo* operon (*nuoA* to *nuoN*). These genes code for all the subunits of a NADH dehydrogenase that is involved in respiratory electron transfer using NADH as an electron donor [31]. The reduced expression of *nuo* in the biofilm organisms may also be related to the organism’s adaptation to low oxygen conditions [32]. The ability of *N. gonorrhoeae* to respire under low oxygen conditions and to form biofilms may offer a survival advantage during cervical infections.

In the present study, we report on a proteomics experiment that compared differential protein expression in biofilm and planktonic organisms using the stable isotope labeling by amino acids in cell culture (SILAC) approach. The SILAC methodology, in which labeled and unlabeled protein solutions are mixed to allow for quantitative analysis by mass spectrometric methods [33], [34], [35], [36], has become a powerful tool for the analysis of differential protein expression in cultured cells. Recently, a few reports have appeared utilizing SILAC for investigations of bacterial cells grown under different physiological conditions [37], [38], [39]. In our experiment, we prepared two parallel populations of *N. gonorrhoeae* consisting of unlabeled biofilm cells and ^13^C_6_-arginine-labeled planktonic cells, and then analyzed mixed protein extracts from these organisms. The differentially expressed proteins that we observed were compared with our transcriptional profiling results [30] and provided further validation of the role of anaerobic respiration in the establishment and maintenance of gonococcal biofilms. Overall, the quantitative proteomic comparisons of biofilm and planktonic *N. gonorrhoeae* emphasized the crucial changes in energy metabolism, outer membrane composition, and nutrient transport that accompany a shift to a biofilm state.

## Materials and Methods

### Materials

The labeled arginine (^13^C_6_-Arg) used in these studies was obtained from Cambridge Isotopes (Andover, MA). Materials related to proteomics, such as sample buffers, 1D and 2D SDS-PAGE gels, and IPG strips (17 cm, pH 3–10) were obtained from Bio-Rad Laboratories (Hercules, CA). The gel stain Coomassie Brilliant Blue R was purchased from Sigma-Aldrich (St. Louis, MO). For proteolysis, sequencing grade, modified trypsin (porcine) was purchased from Promega (Madison, WI). Non-ionic detergent n-dodecyl-β-D-maltoside and additional reagents for protein chemistry including iodoacetamide and dithiothreitol (DTT) were obtained from Sigma-Aldrich (St. Louis, MO). HPLC solvents such as acetonitrile and water were obtained from Burdick & Jackson (Muskegon, MI).

### Bacteria

*N. gonorrhoeae* strain 1291, a piliated clinical isolate that expresses Opa proteins, was used in this study. The strain was reconstituted from frozen stocks as previously described [30]. In this study, the strain was reconstituted onto Morse’s Defined Medium (MDM) [40] plates with either light (L) unlabeled Arg or heavy (H) ^13^C_6_-Arg.

### Growth of Biofilm and Planktonic Populations of *N. gonorrhoeae* with Stable Isotope Labeling

Two separate starter cultures of *N. gonorrhoeae* were grown simultaneously in 15 ml MDM, supplemented with either L-Arg or H-Arg. The starter cultures were inoculated from bacteria grown on MDM plates that were supplemented with the appropriate L- or H-Arg. Cultures were grown at 100 RPM in un-baffled flasks until an O.D._600_ ∼0.25. Approximately 1 ml of the appropriate starter culture was used to inoculate each flow chamber. The cells were allowed to sit in the chambers under static conditions for 1 h at 37°C, after which time a constant flow of the appropriate culture medium (containing either L- or H-Arg) at 150 µl/min was established and allowed to continue for 48 h. The apparatus was equipped with 0.22 µm filters on both the media reservoir and collection flasks to prevent contamination. After 24 h, a sterile glass wool filter was placed in-line after the biofilm growth chambers that were run with H-Arg for the planktonic fraction to filter out any detached biofilm flocs as previously described [30].

### Harvesting of Cells and Preparation of the Three Protein Extracts

From 24–48 h post-inoculation, planktonic cells were collected from the outflow of the biofilm growth chambers, grown using H-Arg supplemented MDM, into a new sterile flask containing 0.1% sodium azide. After 48 h, the biofilm cells were harvested from the chambers grown in L-Arg supplemented MDM. Based upon the relative yields of planktonic and biofilm cells from the biofilm growth apparatus, it was most economical to prepare unlabeled (L) biofilm cells and ^13^C_6_-Arg-labeled (H) planktonic cells. To generate comparable amounts of cells from the two populations, 10 growth chambers were run contemporaneously for biofilm cell collection and 4 growth chambers were run for planktonic cell collection for each biological replicate. The three biological replicates of the experiment were run at different times.

The cells from both planktonic and biofilm preparations were pelleted at 16,000×*g* and the supernatants removed. They were then resuspended in HPLC grade water and repelleted. Finally they were resuspended in HPLC grade water, pelleted and frozen at −80°C until sequential protein extraction was performed using the Bio-Rad Ready Prep Sequential extraction kit that fractionates the proteins into soluble and membrane fractions as follows. Bacteria were rehydrated in 40 mM Tris, sonicated for 8×30 sec with 1 min incubation in ice water between each round of sonication, and then centrifuged at 16,000×*g* for 3 min to produce the soluble protein extract, designated ‘Extract 1’. The insoluble pellet was washed twice in the 40 mM Tris buffer before being extracted in a buffer consisting of 8 M urea, 4% CHAPS, 40 mM Tris, and 0.2% Bio-Lyte 3/10 ampholyte to give ‘Extract 2’ following centrifugation. This second pellet was washed twice in the above buffer before being boiled for 10 min in 2% SDS to give ‘Extract 3’. Protein concentration in Extract 1 was determined using the Bio-Rad Protein assay kit. Protein concentrations for Extracts 2 and 3 were determined using the Bio-Rad DC (detergent-compatible) protein assay kit as described by the manufacturer.

### One-dimensional and Two-dimensional Gel Electrophoresis

For initial pilot experiments, 2D gels were run according to the Bio-Rad Ready Prep 2D kit manual. Briefly, either 200 µg of each (L) or (H) protein fraction, or 100 µg of both, were used to passively rehydrate 17 cm pH 3–10 IPG strips overnight. The strips were then focused in a Bio-Rad PROTEAN IEF system as per manufacturer’s recommendations. After focusing, the IPG strips were equilibrated and run in the second dimension on a 4–12% polyacrylamide gel with an IEF well. The gels were stained/destained using ultrapure HPLC water. The ultrapure water was also used to make the running buffers.

For the 1D SDS-PAGE gels that were used for the majority of the study, equal concentrations of (H) and (L) protein solutions for a given Extract were loaded into the same well of a 4–12% gel. The mixed samples from each Extract were loaded at 40 µg (Extracts 1 and 3) or at 20 µg (Extract 2) on individual gels. The gels were stained with Coomassie and imaged using a ‘Mighty Bright’ UV-light transilluminator (Hoefer, Inc., Holliston, MA) coupled with a Kodak DC-120 camera (Kodak, Rochester, NY). Images were captured and processed with the Kodak 1D Scientific Imaging System software, version 3.6.1.

### In-gel Trypsin Digestion

1D or 2D SDS-PAGE gels stained with Coomassie Blue were placed on a clean glass plate atop a light box. Gel spots (1.5 mm diameter) were manually excised from the gels with a ‘OneTouch’ 2D gel spot picker (Gel Company, San Francisco, CA), placed in a 96-well digester plate, and then covered with 20 µl of HPLC water. For 1D SDS-PAGE gels, contiguous gel bands were excised for each gel lane of between 38 to 48 spots to ensure maximum mass spectrometric coverage of gonococcal proteins. The excised gel pieces were then subjected to digestion with trypsin (Promega, Madison, WI) using a ProGest automatic digester (Genomic Solutions, Ann Arbor, MI). Briefly, the gel spots were destained and dehydrated with acetonitrile. Subsequently, the proteins were reduced with 10 mM DTT at 60°C for 30 min, alkylated with 100 mM iodoacetamide (37°C, 45 min) and then incubated with 125–250 ng sequencing grade trypsin at 37°C for 4 h. The resulting tryptic peptides were then extracted from the gel by aqueous/10% formic acid extraction, adjusted to a volume of approximately 10–15 µl each, and analyzed by mass spectrometry.

### MALDI-TOF MS Analysis

For preliminary analysis of selected peptide digestions, samples were analyzed by matrix-assisted laser desorption/ionization time-of-flight (MALDI-TOF) mass spectrometry on a Voyager DE-STR plus mass spectrometer (AB Sciex, Concord, Canada) operating in the positive-ion reflectron mode with delayed extraction. The accelerating voltage was 25 kV, the grid was set at 71% of full accelerating voltage, and the delay time was 275 ns. Samples were mixed 1∶1 with a matrix solution of α-cyano-4-hydroxycinnamic acid in acetonitrile/MeOH (Agilent Technologies, Palo Alto, CA) and 1 µl was spotted onto a stainless steel target. Typically, ∼100 laser shots were acquired and the spectra were externally calibrated with a mixture consisting of angiotensin I, and ACTH fragments 1–17, 18–39, and 7–38 (Bachem, Torrance, CA).

### Nano-LC-MS/MS Analysis

All proteolytic peptide digests were analyzed by reverse-phase nano-HPLC-MS/MS. Briefly, peptides were separated on an Ultimate nanocapillary HPLC system equipped with a PepMap™ C18 nano-column (75 µm I.D. ×15 cm; Dionex, Sunnyvale, CA) and CapTrap Micro guard column (0.5 µl bed volume; Michrom, Auburn, CA). Peptide mixtures were loaded onto the guard column and washed with the loading solvent (H_2_O/0.05% formic acid, 20 µl/min) for 5 min, then transferred onto the analytical C18-nanocapillary HPLC column and eluted at a flow rate of 300 nl/min using the following gradient: 2% B (from 0–5 min), and 2–70% B (from 5–55 min). Solvent A consisted of 0.05% formic acid in 98% H_2_O/2% acetonitrile and solvent B consisted of 0.05% formic acid in 98% acetonitrile/2% H_2_O. The column eluant was directly coupled to a ‘QSTAR Pulsar i’ quadrupole orthogonal TOF mass spectrometer (MDS Sciex, Concorde, Canada) equipped with a Protana/ProXeon nanospray ion source (ProXeon Biosystems, Odense, Denmark). The nanospray needle voltage was typically 2300 V in the HPLC-MS mode. Electrospray ionization mass spectra (ESI-MS) and tandem mass spectra (ESI-MS/MS) were recorded in positive-ion mode with a resolution of 12,000–15,000 FWHM. For collision induced dissociation tandem mass spectrometry, the mass window for precursor ion selection of the quadrupole mass analyzer was set to ±1 *m/z*. The precursor ions were fragmented in a collision cell using nitrogen as the collision gas. Spectra were calibrated in static nanospray mode using MS/MS fragment ions of a renin peptide standard (His immonium ion with *m/z* at 110.0713 and b_8_-ion with *m/z* at 1028.5312).

### Identification and Quantification of *N. gonorrhoeae* Proteins

The ESI-MS/MS spectra were analyzed using ProteinPilot 4.0 software (revision 148085) running the Paragon Algorithm 4.0.0.0, 148083 developed by AB Sciex [41]. The software generates peak lists that were searched against a *N. gonorrhoeae* custom database downloaded from the Kyoto Encyclopedia of Genes and Genomes (KEGG) website (http://www.genome.jp/kegg/) consisting of 2002 protein sequences from *N. gonorrhoeae* strain FA 1090 (file dated 05/17/2010). For quantitative analysis of the SILAC results, all MS/MS spectra from fractions from a single 1D gel (i.e., from a single Extract) were submitted as a batch for data processing. The following search parameters were used: sample type was defined as ‘SILAC (Arg+6)’, iodoacetamide was selected as our Cys alkylation method, enzyme specificity was defined as trypsin, QSTAR ESI was indicated as instrument type, and ‘gel-based ID’ was selected as an additional parameter. Under processing specifications, the ‘quantitate’ and ‘bias correction’ features were engaged and our ID focus was specified as ‘biological modifications’, allowing for a range of amino acid variable modifications to be considered. The search effort was set to ‘Thorough ID’ and the False Discovery Rate Analysis was engaged, with the default setting for the ‘Detected Protein Threshold [Unused ProtScore (Conf)]’ at >0.05 (10.0%) to allow for the most accurate determination of error rates. Additionally, we made two modifications to the ProteinPilot 4.0 default parameters in the ‘ProteinPilot.exe.config’ file: the ‘Auto Discordant Peptide Threshold’ value was set to 0.0 and the ‘Confidence Percent Threshold For Including Self In Quant’ value was set to 50. This latter parameter restricted the MS scans used to derive L:H ratios to only those associated with peptides identified with a confidence score of at least 50%.

The mass tolerance for data generated from the QSTAR pulsar i was considered <100 ppm both on the precursor and fragment ion level. In addition, ProteinPilot 4.0 performs a mass recalibration of the datasets during the search based on highly confident peptide search results. Specifically, a first search iteration is done to identify high confidence peptides that are then used to recalibrate both the MS and MS/MS spectra. The recalibrated data is then automatically re-searched. In contrast to other search engines, the Paragon Algorithm uses probabilities rather than specified settings to assess features such as modifications, substitutions, and cleavage events. In conducting the quantification, the Paragon Algorithm derives a bias factor for the dataset to correct for imperfect 1∶1 mixing. The reported protein L:H ratios are normalized with this factor, allowing for direct comparisons between datasets. A detailed description of how the Paragon Algorithm differs from conventional search engines has been published [41].

To assess and restrict rates of false positive peptide/protein identifications, we used the Proteomics System Performance Evaluation Pipeline (PSPEP) tool available in the ProteinPilot 4.0 software package. This tool automatically creates a concatenated forward and reverse decoy database to search against, and provides a Microsoft Excel output of the experimentally determined false discovery rate at the spectral, peptide and protein levels [42]. In the present study, we included in our datasets only proteins with an ‘Unused ProtScore’ of ≥2.0, which corresponds to a protein confidence cut-off threshold of 99%. At this protein confidence threshold, the protein global false discovery rate (FDR) from fit of the data was 0.02% or lower in all of the proteomics datasets.

For inspection and output of our quantitative SILAC results, all protein ratios were expressed as L:H to give biofilm/planktonic ratios. Only quantified proteins with good p-values (<0.05) were included in our sets of differentially expressed proteins from each Extract. To further verify these results, the MS spectra from peptides used in the quantification of all proteins given a p-value by the software (p-values ranging from 0–1), were manually inspected. Spectra were removed from use in the quantification if they exhibited any of the following problems: (1) interference from overlapping peptide signals, (2) signal to noise ratio of <4 for the largest of the SILAC partners, (3) poor isotope pattern for both of the SILAC partners, (4) sequence assignment involving unlikely modifications, especially when associated with an outlying ratio measurement, (5) obvious quantitative measurement errors generally associated with incorrect software assignment of extreme ratios, and (6) obvious outlying measurements associated with a peptide included from an outlying LC-MS/MS file. In addition to these criteria, only quantified proteins whose identifications were based on at least 2 peptides at the 95% peptide confidence level were included in the final set of quantified proteins. Imposition of this latter additional criterion resulted in the removal of only one measurement from one dataset in the study.

### Compilation of Proteomic Results

To compile results from all of the individual proteomics datasets, an in-house computer program developed at UCSF was used to sort entries by protein accession numbers and generate Excel spreadsheets. The program reads in the ProteinPilot search engine ‘Protein Summary Results’ and ‘Peptide Summary Results’ files to create a table of protein entries (all with Unused ProtScores of ≥2.0) with lists of associated peptides used in the identification and quantification of each protein. Columns in the spreadsheet contain entries from each Extract from each of the three biological replicates. The program can be set to filter the raw results to include only quantified proteins with quantitation p-values <0.05. Additionally, to allow for averaging of repeat measurements by Extracts across the biological replicates of the experiment, the raw protein L:H ratios can be output as log_2_ values. From the sorted spreadsheet, a table of average protein ratios (log_2_ values) for all of the quantified proteins with p-values <0.05 was compiled. If a protein was detected in more than one Extract, its average value was computed in each of the Extracts it was measured in.

The functional role assignments (both main and sub-roles) indicated for the *N. gonorrhoeae* proteins were downloaded from the J. Craig Venter Institute-Comprehensive Microbial Resource (JCVI-CMR) website (http://cmr.jcvi.org/tigr-scripts/CMR/CmrHomePage.cgi). A small number of proteins whose functional role assignments were not available from the JCVI-CMR website were given tentative assignments after conducting BLAST and KEGG orthology searches to identify possible orthologs, whose functional role assignments were then obtained from the JCVI-CMR website. In all cases, proteins from the related pathogen *N. meningitidis* were among those with the highest % identity to the *N. gonorrhoeae* proteins in question and were selected to provide the tentative functional role assignments. Predicted subcellular locations for the *N. gonorrhoeae* proteins determined by PSORTb v. 3.0 were also downloaded (http://www.psort.org/). Additionally, we combined our proteomics results with the list of differentially expressed genes from our previous study of transcriptional profiling of gonococcal biofilms [30] and submitted the combined hits to the KEGG website (http://www.genome.jp/kegg/) for mapping onto KEGG biosynthetic pathways.

## Results

### *N. gonorrhoeae* Planktonic and Biofilm Growth with Stable Isotope Labeling

*N. gonorrhoeae* strain 1291 is an arginine auxotroph. At the beginning of this study, we demonstrated that ^13^C_6_-arginine could be readily taken up by *N. gonorrhoeae* and incorporated into proteins without isotopic scrambling. Protein digests from in-gel digestion of several gel bands from organisms grown under ^13^C_6_-Arg supplementation were analyzed by MALDI-TOF MS and showed an incorporation efficiency of ≥97%.

**Figure 1:**
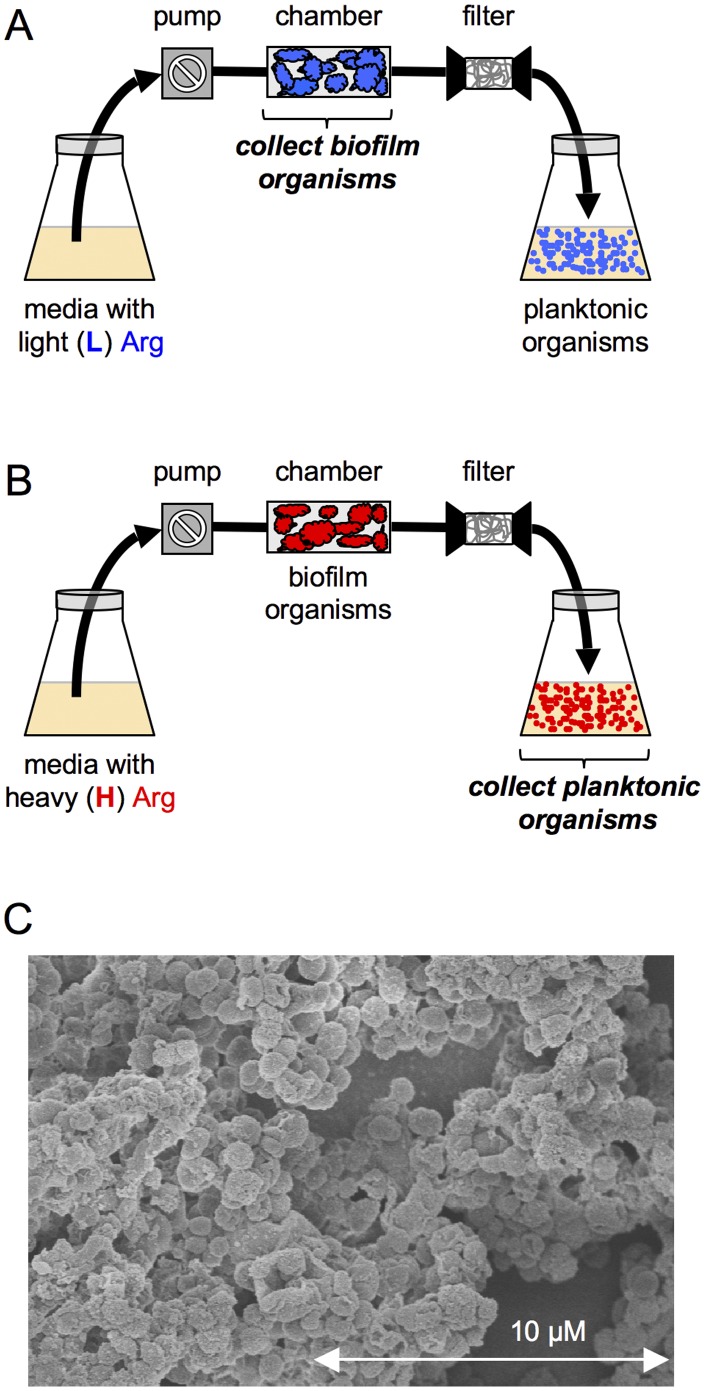
Schematic illustrations of the continuous-flow apparatus used in the production of biofilm and planktonic populations of *N. gonorrhoeae*. (A) Biofilm organisms were collected from experiments using light (L) unlabeled arginine added to Morse’s Defined Medium and (B) planktonic organisms were collected from experiments using heavy (H) ^13^C_6_-arginine added to Morse’s Defined Medium. The collection of planktonic organisms was begun 24 h post-inoculation; both planktonic and biofilm organisms were harvested 48 h post-inoculation. (C) Scanning electron microscope image of *N. gonorrhoeae* biofilm growing on a glass bead.

A continuous-flow apparatus illustrated in Figure 1 was used for the production of *N. gonorrhoeae* biofilms and the collection of planktonic cells from the biofilm outflow. To generate samples designed for the SILAC experiment employing arginine [43], we prepared two parallel populations of *N. gonorrhoeae*, the unlabeled biofilm cells (Figure 1A) and the ^13^C_6_-Arg labeled planktonic cells (Figure 1B). In a typical 48 h experiment, approximately 10^9^ – 10^10^ cfu of biofilm cells and 10^9^ – 10^10^ cfu of planktonic cells were obtained.

**Figure 2:**
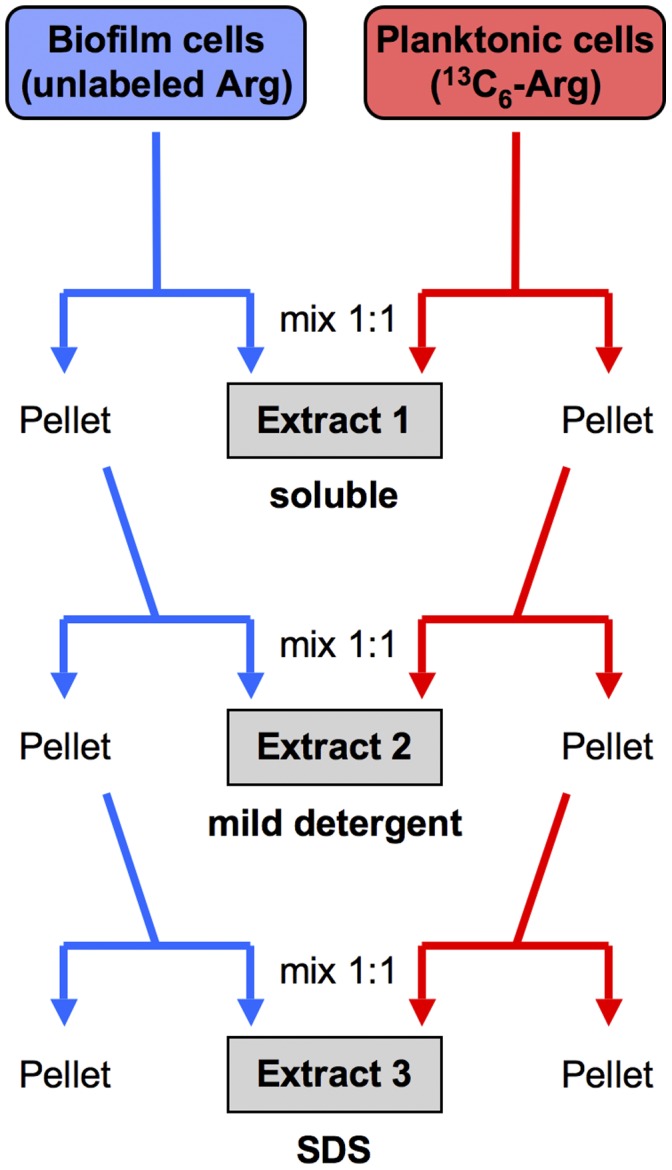
SILAC mixing scheme. The three protein extracts were prepared separately from the biofilm and planktonic organisms, and then mixed 1∶1 by protein concentration. The extraction conditions were as follows: **Extract 1,** sonication in 40 mM Tris; **Extract 2,** extraction in 8 M urea, 4% Chaps, 40 mM Tris, and 0.2% Bio-Lyte 3/10 ampholyte; and **Extract 3,** boiling for 10 min in 2% SDS.

The biofilm and planktonic cells were then independently taken through three extraction steps to generate three separate protein extracts (Extracts 1–3) to both decrease the sample complexity for MS analysis and ensure coverage of even the most hydrophobic proteins (see Figure 2). The first extraction condition (sonication in 40 mM Tris buffer) released soluble proteins to give Extract 1. The subsequent extraction conditions were designed to solubilize membrane proteins (Extracts 2 and 3). On the basis of measured protein concentrations, the corresponding unlabeled (L) biofilm and ^13^C_6_-Arg-labeled (H) planktonic Extracts were mixed 1∶1 as shown in Figure 2. Thus, three mixed protein Extracts (L + H) were generated from one biological experiment. The entire protocol was repeated on three different occasions to give three biological replicates.

### Preliminary Analysis (Pilot Experiment) of Soluble Proteins Using 2D SDS-PAGE

Prior to analyzing the three extracts, a pilot experiment was conducted to assess the suitability of the overall experimental design. In this experiment, the soluble proteins (Extract 1) from biofilm and planktonic cells were run individually on 2D SDS-PAGE gels to obtain an initial overview of protein expression between biofilm and planktonic populations of *N. gonorrhoeae*. When compared side by side as shown in Supplementary Figure S1 (panels A and B), there were a few obvious spot differences between the two gels, suggestive of differential protein expression. To enable the quantification of protein expression changes using the SILAC MS experiment [33], [34], [35], [36], a 2D SDS-PAGE gel of the mixed protein Extract (L + H) was also run, from which 55 spots were excised and subjected to tryptic digestion (see Supplementary Figure S1C). Following analysis by MALDI-TOF MS, selected samples were analyzed by HPLC-MS/MS.

For protein identification and quantification, a ProteinPilot search of the LC-MS/MS data was carried out against a custom database of 2002 *N. gonorrhoeae* proteins listed in KEGG. In this preliminary experiment, 91 proteins were identified. Of these proteins, 15 showed differential expression with p-values <0.05. Supplementary Table S1 lists these 8 upregulated and 7 downregulated proteins. As anticipated, the two most highly upregulated proteins, NGO0574 (carbonic anhydrase, Cah) and NGO1812 (major outer membrane protein porin P.IB), as well as the two most highly downregulated proteins, NGO0108 (hypothetical protein) and NGO1871 (peptide deformylase), were derived from 2D gel spots with visible differences in the comparison gels (see Supplementary Figure S1). As will be presented in the subsequent section, these proteins were found to constitute a small subset of the total proteins identified in our final optimized protocol that used a combined 1D gel separation and HPLC-MS/MS approach.

### Differential Protein Expression in Soluble and Membrane Protein Fractions

**Figure 3:**
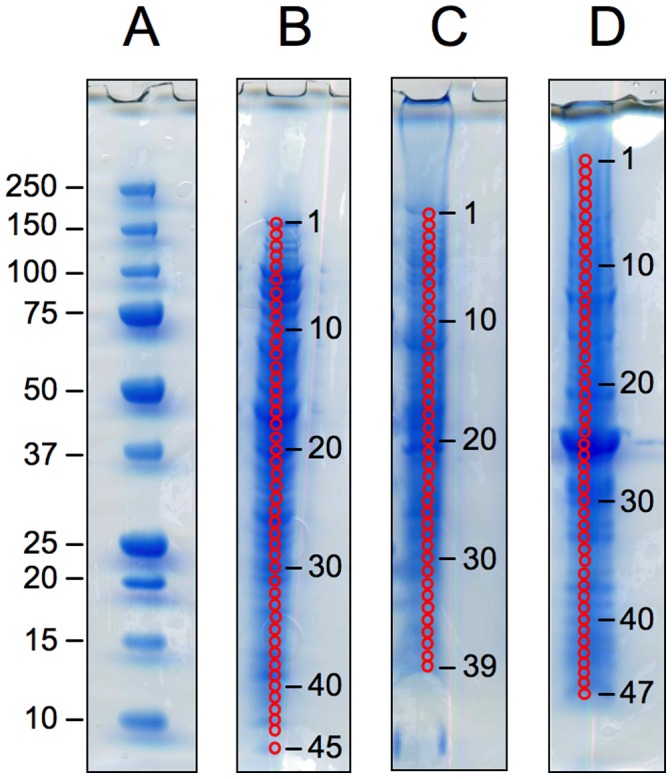
1D SDS-PAGE gels of mixed protein extracts (biofilm + planktonic) from one representative biological replicate. The four gels show (A) molecular weight markers, with molecular weights given in KDa, (B) the Extract 1 protein mixture, 40 µg loaded, (C) the Extract 2 protein mixture, 20 µg loaded, and (D) the Extract 3 protein mixture, 40 µg loaded. Bands were excised from the gel lanes as indicated by the numbered red circles.

While the 2D SDS-PAGE gel approach allowed us to initially assess overall protein expression changes and to establish incorporation efficiency of the heavy labeled ^13^C_6_-Arg, for a more comprehensive, in-depth investigation of the *N. gonorrhoeae* proteome, we subsequently chose a 1D SDS-PAGE approach. This approach also allowed better separation and proteomic coverage of membrane proteins, as 2D gels are known to be problematic with regard to hydrophobic proteins. As shown in Figure 3, Extracts 1, 2, and 3 from each biological replicate were separated on 1D SDS-PAGE gels and ∼40–45 bands were excised contiguously from each gel lane. Following in-gel digestion with trypsin, proteolytic peptide mixtures obtained from each band were subjected to HPLC-MS/MS analysis.

Across the three biological replicates of the experiment, a total of 757 unique *N. gonorrhoeae* proteins were identified in the ProteinPilot searches (see Supplementary Table S2). Of these identified proteins, 231 proteins were measured as differentially expressed at least once in the study, with p-values <0.05 (see Supplementary Table S2 for tabulated quantitative results; Supplementary Table S3 for full protein level information on quantified proteins; and Supplementary Tables S4A, S4B, and S4C for full peptide level information on quantified proteins from Extracts, 1, 2, and 3, respectively). After conversion to log_2_ values, repeat quantitative measurements were averaged for each extract. With the application of a ≥1.5-fold cutoff threshold for differential expression, a total of 152 proteins were characterized as significantly differentially expressed between the biofilm and planktonic states. This number represented approximately 7.6% of the *N. gonorrhoeae* proteome.

**Figure 4:**
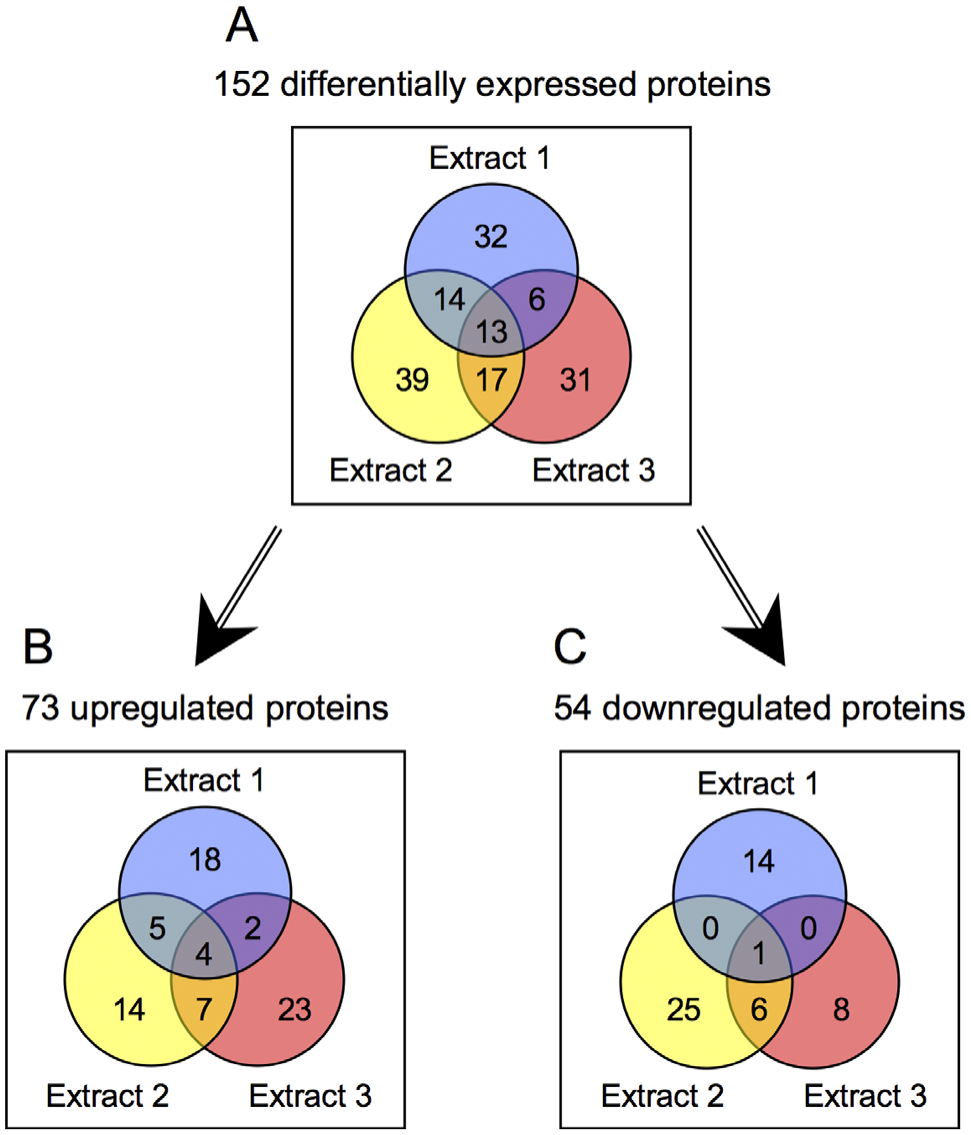
Venn diagrams showing the numbers of differentially expressed proteins identified in Extracts 1–3 over the three biological replicates of the experiment, comparing biofilm to planktonic organisms. The three extraction conditions progressed from the least stringent (Extract 1, 30 mM Tris buffer) to the most stringent (Extract 3, 2% SDS) so as to capture as many different classes of proteins as possible. All proteins represented had quantitation p-values <0.05 and met the 1.5-fold cutoff threshold for differential expression. The diagrams show (A) all differentially expressed proteins, (B) upregulated proteins, and (C) downregulated proteins. Twenty-five of the 152 proteins represented in panel A exhibited variable expression trends across Extracts (see text).

The Venn diagram in Figure 4A shows the distribution of all 152 differentially expressed proteins according to the Extract(s) in which they were detected. The diagrams in Figures 4B and 4C separate out the 73 upregulated and 54 downregulated proteins, respectively. As indicated, a significant number of differentially expressed proteins (102 of 152) were detected in single extracts, whereas only 50 proteins were detected in multiple extracts. Of those proteins found in multiple extracts, 25 showed different expression trends in different extracts and were classified as variable. Complete listings of the upregulated, downregulated, and variable proteins are given in Supplementary Tables S5, S6, and S7, respectively.

### Classification of Differentially Expressed Proteins by Functional Roles

**Figure 5:**
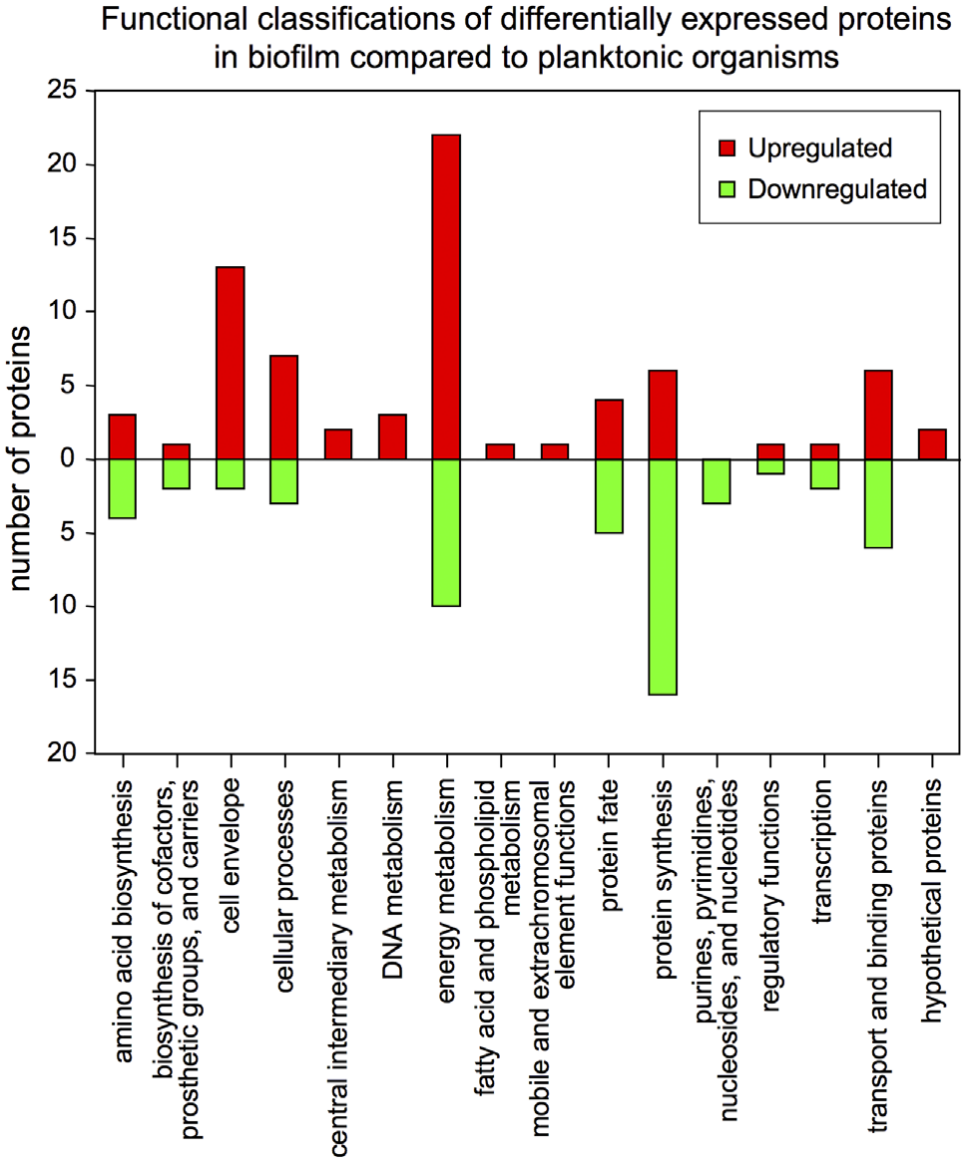
Plot of the functional classifications of all upregulated and downregulated proteins in the biofilm compared to the planktonic growth form. All of the tabulated proteins met a 1.5-fold cutoff threshold for differential expression, with quantitation p-values <0.05. Functional role assignments for the *N. gonorrhoeae* proteins were downloaded from the JCVI-CMR website (http://cmr.jcvi.org/tigr-scripts/CMR/CmrHomePage.cgi). In a few cases where functional role assignments were not available, the *N. gonorrhoeae* proteins were given tentative assignments in the present study after conducting BLAST and KEGG orthology searches (see Supplementary Tables S5 and S6 for details).

Known functional roles (both main and sub-roles) for most of the *N. gonorrhoeae* proteins were available from the JCVI-CMR website (http://cmr.jcvi.org/tigr-scripts/CMR/CmrHomePage.cgi), and this information is given in Supplementary Tables S5, S6, and S7. Figure 5 shows a plot of the main functional roles of all of the upregulated and downregulated proteins that met an average fold change cutoff threshold of ≥1.5. As indicated, the major functional categories of upregulated proteins included cell envelope, cellular processes, energy metabolism, protein synthesis, and transport and binding proteins. The main functional categories of downregulated proteins were energy metabolism, protein fate, protein synthesis, and transport and binding proteins.

Of the 73 upregulated proteins listed in Supplementary Table S5, 43 proteins were detected as upregulated more than 2-fold (log_2_ values >1) at least once, including 12 proteins showing more than a 3-fold increase (log_2_ values >1.585). This set of the most highly upregulated proteins is given in Table 1 according to functional roles. Likewise, a high percentage of the downregulated proteins listed in Supplementary Table S6 (21 of 54) were downregulated more than 2-fold at least once, including 10 highly downregulated proteins that exhibited more than a 3-fold decrease. These highly downregulated proteins are given in Table 2.

**Table 1:** Upregulated proteins in *N. gonorrhoeae* biofilms listed by functional roles^a^: Biofilm/Planktonic ratios expressed as log_2_ values, 2-fold increase (log_2_ values ≥1.000) measured in at least one Extract, and p-values <0.05.

			Extract 1		Extract 2		Extract 3	
Accession	Name	JCVI sub-role	Avg^b^	(N)^c^	Avg^b^	(N)^c^	Avg^b^	(N)^c^
**Amino acid biosynthesis**								
NGO1242	HisB; imidazoleglycerol-phosphate dehydratase	Histidine family	1.331	(1)				
NGO0340	putative cysteine synthase/cystathionine beta-synthase	Serine family			1.378	(3)	0.816	(1)
**Cell envelope**								
NGO1513	OpaD	Other	2.018	(2)	1.552	(2)	2.549	(2)
NGO0055	pilus-associated protein	Surface structures	0.806	(2)	1.324	(1)		
NGO1669	PilG; type IV pilus assembly protein PilC	Surface structures			1.276	(1)	0.644	(2)
NGO0233	outer membrane protein	Other			1.038	(2)	1.093	(3)
NGO0070	outer membrane opacity protein B	Other					2.379	(1)
NGO1949^e^	hypothetical protein	Other					1.739	(1)
NGO0948^e^	hypothetical protein; lipoprotein-34	Other					1.222	(3)
NGO1801	hypothetical protein	Other					1.199	(1)
NGO0094^d^	hypothetical protein; type IV pilus assembly protein PilQ	Surface structures					1.098	(3)
NGO1251^e^	hypothetical protein	Other					1.045	(1)
**Cellular processes**								
NGO1225	putative peptidyl-prolylisomerase	Pathogenesis	1.387	(1)				
NGO1382	putative GTP pyrophosphokinase	Adaptations to atypical conditions			1.348	(1)		
NGO0382	hypothetical protein; cell division protease FtsH	Cell division					1.071	(1)
NGO0277	ComL; putative lipoprotein	DNA transformation					1.031	(2)
**Central intermediary metabolism**								
NGO1276	AniA; nitrite reductase (NO-forming)	Nitrogen metabolism	3.485	(1)	3.175	(1)	1.742	(1)
NGO0574	Cah; carbonic anhydrase	Other	1.525	(3)	0.668	(2)		
**Energy metabolism**								
NGO1769	CcpR; cytochrome c peroxidase	Electron transport	3.271	(1)	1.547	(2)		
NGO0564	dihydrolipoamide acetyltransferase	Pyruvate dehydrogenase	1.724	(3)				
NGO1812^d^	major outer membrane protein porin P.IB	Electron transport	1.669	(3)	0.908	(3)	0.697	(3)
NGO2146	F0F1 ATP synthase subunit B; F-type H+-transporting ATPase subunit b	ATP-proton motive force interconversion	1.637	(2)	0.717	(2)	0.593	(3)
NGO0906	hypothetical protein	Electron transport	1.073	(1)	0.614	(1)		
NGO1373	cbb3-type cytochrome c oxidase subunit II	Electron transport			1.479	(1)	1.930	(1)
NGO0375	Pgm; phosphoglucomutase	Sugars			1.368	(1)		
NGO0718^d^	RpiR family transcriptional regulator	Glycolysis/gluconeogenesis			1.217	(1)		
NGO0214	putative phosphotransacetylase	Fermentation			1.177	(2)		
NGO1328	putative cytochrome	Electron transport			1.152	(1)		
NGO1371	CcoP; cb-type cytochrome c oxidase subunit III	Electron transport					1.602	(3)
NGO1985^e^	hypothetical protein	Electron transport					1.351	(3)
NGO1470	PntA; NAD(P) transhydrogenase subunit alpha	Electron transport					1.293	(1)
NGO1584^e^	MafA3; putative adhesin	Electron transport					1.255	(2)
NGO2031	PetC; ubiquinol-cytochrome c reductase cytochrome c1 subunit	Electron transport					1.233	(3)
**Protein fate**								
NGO0399	heat shock protein HtpX	Protein folding and stabilization			1.668	(1)	1.683	(1)
**Protein synthesis**								
NGO1844	30S ribosomal protein S7	Ribosomal proteins: synthesis and modification	1.219	(1)				
NGO1832	RpsC; 30S ribosomal protein S3	Ribosomal proteins: synthesis and modification			1.325	(3)		
NGO1830^e^	RpsQ; 30S ribosomal protein S17						1.120	(3)
**Transport and binding proteins**								
NGO0206	putative ABC transporter, periplasmic binding protein, polyamine	Amino acids, peptides and amines	1.217	(1)				
NGO0372	putative ABC transporter, periplasmic binding protein, amino acid	Cations and iron carrying compounds	1.109	(1)			0.609	(1)
NGO0794	BfrA; bacterioferritin	Cations and iron carrying compounds	1.090	(2)				
NGO0455	hypothetical protein; type IV pilus assembly protein PilX	Unknown substrate					1.784	(1)
NGO1205	putative TonB-dependent receptor protein; iron complex outermembrane receptor protein	Cations and iron carrying compounds					1.530	(2)
**Hypothetical proteins**								
NGO0236	hypothetical protein	Conserved					1.344	(1)

a Functional role assignments (both main and JCVI sub-roles) for the *N. gonorrhoeae* proteins were downloaded from the JCVI-CMR website.

b Average log_2_ values for biofilm/planktonic protein ratios.

c (N) represents the number of measurements (biological replicates) averaged.

d Proteins with more than one functional role assignment provided (Main role/JCVI sub-role): NGO0094, Cellular processes/DNA transformation, Cellular processes/Pathogenesis; NGO1812, Energy metabolism/Fermentation; NGO0718, Energy metabolism/Sugars.

e Protein functional role category tentatively assigned in the present study by orthology to an *N. meningitidis* protein (see Supplementary Table S5 for details).

**Table 2:** Downregulated proteins in *N. gonorrhoeae* biofilms listed by functional roles^a^: Biofilm/Planktonic ratios expressed as log_2_ values, 2-fold decrease (log_2_ values ≤−1.000) measured in at least one Extract, and p-values <0.05.

			Extract 1		Extract 2		Extract 3	
Accession	Name	JCVI sub-role	Avg^b^	(N)^c^	Avg^b^	(N)^c^	Avg^b^	(N)^c^
**Amino acid biosynthesis**								
NGO0947	DapA; dihydrodipicolinate synthase	Aspartate family			−1.901	(1)		
NGO1961	argininosuccinate synthase	Glutamate family			−1.643	(1)		
NGO0397	HisZ; ATP phosphoribosyltransferase regulatory subunit	Histidine family			−1.320	(1)		
**Biosynthesis of cofactors, prosthetic groups, and carriers**								
NGO0704	bifunctional 3,4-dihydroxy-2-butanone 4-phosphate synthase/GTP cyclohydrolase II-like protein	Riboflavin, FMN, and FAD	−1.100	(1)				
NGO1684	7-cyano-7-deazaguanine reductase	Folic acid			−1.210	(1)		
**Energy metabolism**								
NGO2149	F0F1 ATP synthase subunit gamma; F-type H+-transporting ATPase subunit gamma	ATP-proton motive force interconversion			−2.138	(1)		
NGO2148	F0F1 ATP synthase subunit alpha; F-type H+-transporting ATPase subunit alpha	ATP-proton motive force interconversion			−1.392	(3)	−1.621	(1)
NGO0639^d^	putative L-lactate dehydrogenase; L-lactate dehydrogenase (cytochrome)	Anaerobic			−1.096	(3)	−0.849	(3)
NGO0565	AceE; pyruvate dehydrogenase subunit E1	Pyruvate dehydrogenase			−0.875	(3)	−1.178	(3)
**Protein fate**								
NGO1422	heat shock protein GrpE; molecular chaperone GrpE	Protein folding and stabilization			−2.206	(1)		
NGO1046	putative ClpB protein; ATP-dependent Clp protease ATP-binding subunit ClpB	Degradation of proteins, peptides, and glycopeptides			−1.798	(1)		
NGO0116	preprotein translocase subunit SecB	Protein and peptide secretion and trafficking			−1.572	(1)		
NGO2095	GroEL; chaperonin GroEL	Protein folding and stabilization			−1.528	(3)	−1.875	(2)
**Protein synthesis**								
NGO0174	RpsP; 30S ribosomal protein S16	Ribosomal proteins: synthesis and modification			−1.579	(1)		
NGO0304	PheT; phenylalanyl-tRNA synthetase subunit beta	tRNA aminoacylation			−1.434	(1)		
NGO1843	FusA; elongation factor G	Translation factors			−1.381	(1)		
NGO1838	RplC; 50S ribosomal protein L3	Ribosomal proteins: synthesis and modification			−1.297	(2)		
NGO2024	RplM; 50S ribosomal protein L13	Ribosomal proteins: synthesis and modification			−1.123	(1)		
**Transport and binding proteins**								
NGO2093	FetA; iron complex outermembrane receptor protein	Cations and iron carrying compounds	−2.447	(1)	−2.440	(2)	−2.008	(3)
NGO1496^e^	TbpB; transferrin-binding protein B	Cations and iron carrying compounds			−2.418	(2)		
NGO1495	TbpA; transferrin-binding protein A	Cations and iron carrying compounds			−0.680	(1)	−1.623	(2)

a Functional role assignments (both main and JCVI sub-roles) for the *N. gonorrhoeae* proteins were downloaded from the JCVI-CMR website.

b Average log_2_ values for biofilm/planktonic protein ratios.

c (N) represents the number of measurements (biological replicates) averaged.

d Protein with more than one functional role assignment provided (Main role/JCVI sub-role): NGO0639, Energy metabolism/Glycolysis/gluconeogenesis.

e Protein functional role category tentatively assigned in the present study by orthology to an *N. meningitidis* protein (see Supplementary Table S6 for details).

Fifteen of the 43 proteins measured as upregulated more than 2-fold were involved in energy metabolism, including 6 proteins that were upregulated more than 3-fold (Table 1). Strong protein hits with replicate measurements in this category include cytochrome c peroxidase (CcpR), dihydrolipoamide acetyltransferase, major outer membrane protein porin P.IB, F0F1 ATP synthase subunit B, cbb3-type cytochrome c oxidase subunit II, and cb-type cytochrome c oxidase subunit III (CcoP). The next largest group of highly upregulated proteins (10 proteins) was cell envelope proteins. This group included three outer membrane proteins (OpaD, outer membrane protein NGO0233, and outer membrane opacity protein B), one pilus-associated protein (NGO0055), and two pilus assembly proteins (PilG and PilQ). Of the 5 upregulated proteins classified as transport and binding proteins, bacterioferritin (BfrA), a putative TonB-dependent receptor protein (iron complex outermembrane receptor protein), and a putative periplasmic binding protein of an amino acid ABC transporter were all found to be upregulated in multiple extracts. NGO0340, a putative cysteine synthase/cystathionine beta-synthase involved in amino acid biosynthesis, was another highly upregulated protein that was identified repeatedly. Finally, in the category of central intermediary metabolism, nitrite reductase (AniA) and carbonic anhydrase (Cah) were key upregulated proteins. As seen in Table 1, the four most significantly upregulated proteins exhibiting expression increases in the range of 4- to 11-fold (log_2_ 2–3.5) were AniA, CcpR, outer membrane opacity protein B, and OpaD.

Proteins that met a 2-fold cutoff threshold for downregulation in biofilm populations included categories of energy metabolism (4 proteins), protein fate (4 proteins), protein synthesis (5 proteins), and transport and binding proteins (3 proteins), some of which were among the most highly downregulated proteins observed in this study. The three transport and binding proteins, iron complex outermembrane receptor protein (FetA), transferrin-binding protein B (TbpB), and transferrin-binding protein A (TbpA), were repeatedly measured with high fold changes (see Table 2). In fact, FetA was the most highly downregulated protein detected in this study, showing a 4 to 5.5-fold decrease (log_2_ values in the range of −2 to −2.5) in multiple extracts. Additionally, TbpB was also found to be downregulated ∼5-fold. Of the highly downregulated proteins involved in energy metabolism, three were measured repeatedly: F0F1 ATP synthase subunit alpha, putative L-lactate dehydrogenase, and pyruvate dehydrogenase subunit E1 (AceE). Of the hits involved in protein fate, the heat shock protein GrpE was most highly downregulated, and chaperonin GroEL was measured repeatedly. Most of the proteins involved in protein synthesis were ribosomal proteins; however the 50S ribosomal protein L3 (RplC) was the only protein in this category that showed significant differences between the biofilm and planktonic cells more than once.

### Differential Expression of Outer Membrane Proteins

In addition to categorizing differentially expressed proteins by their functional role assignments, we also evaluated the predicted subcellular localizations of proteins as given by the PSORTb program (http://www.psort.org/). Table 3 gives a complete listing of the 37 predicted outer membrane proteins for *N. gonorrhoeae* FA 1090. In our proteomics study, 23 of these predicted proteins (62%) were observed, with 15 designated as differentially expressed (≥1.5-fold change, with p-values <0.05). While some of these differentially expressed outer membrane proteins could be detected in the soluble fraction (Extract 1), many of them were only found in Extracts 2 and/or 3.

**Table 3:** Predicted outer membrane proteins in *N. gonorrhoeae* determined by PSORTb [62], [63].^a^.

Expression^b^	Accession	Protein Name	Final Localization	Final Score	Accession
up	NGO0055	pilus-associated protein	Outer Membrane	9.49	gi|59800520|ref|YP_207232.1|
up	NGO0070	outer membrane opacity protein B	Outer Membrane	9.99	gi|59800533|ref|YP_207245.1|
up	NGO0094	hypothetical protein; type IV pilus assembly protein PilQ	Outer Membrane	9.99	gi|59800555|ref|YP_207267.1|
up	NGO0233	outer membrane protein	Outer Membrane	9.99	gi|59800686|ref|YP_207398.1|
up	NGO0277	ComL; putative lipoprotein	Outer Membrane	9.93	gi|59800727|ref|YP_207439.1|
up	NGO1225	putative peptidyl-prolylisomerase	Outer Membrane	9.93	gi|59801584|ref|YP_208296.1|
up	NGO1363	hypothetical protein	Outer Membrane	9.99	gi|59801711|ref|YP_208423.1|
up	NGO1513	OpaD	Outer Membrane	9.99	gi|59801851|ref|YP_208563.1|
up	NGO1577	Omp3	Outer Membrane	9.99	gi|59801906|ref|YP_208618.1|
up	NGO1801	hypothetical protein	Outer Membrane	9.99	gi|59802119|ref|YP_208831.1|
up	NGO1812	major outer membrane protein porin P.IB	Outer Membrane	9.99	gi|59802130|ref|YP_208842.1|
down	NGO1495	TbpA; transferrin-binding protein A	Outer Membrane	9.99	gi|59801833|ref|YP_208545.1|
down	NGO2093	FetA; iron complex outermembrane receptor protein	Outer Membrane	9.99	gi|59802394|ref|YP_209106.1|
variable	NGO1056	hypothetical protein; lipoprotein NlpD	Outer Membrane	9.92	gi|59801427|ref|YP_208139.1|
variable	NGO1656	hypothetical protein	Outer Membrane	9.93	gi|59801983|ref|YP_208695.1|
D	NGO0595	hypothetical protein; type IV pilus assembly protein PilF	Outer Membrane	9.92	gi|59801025|ref|YP_207737.1|
D	NGO0994	Laz	Outer Membrane	9.99	gi|59801378|ref|YP_208090.1|
D	NGO1492	putative phospholipase; phospholipase A1	Outer Membrane	9.99	gi|59801830|ref|YP_208542.1|
D	NGO1715	OstA; LPS-assembly protein	Outer Membrane	9.99	gi|59802036|ref|YP_208748.1|
D	NGO1780	hypothetical protein	Outer Membrane	9.93	gi|59802099|ref|YP_208811.1|
D	NGO1956	hypothetical protein	Outer Membrane	9.99	gi|59802267|ref|YP_208979.1|
D	NGO2048	GNA33); membrane-bound lytic murein transglycosylase A	Outer Membrane	9.92	gi|59802356|ref|YP_209068.1|
D	NGO2121	hypothetical protein; lipoprotein	Outer Membrane	9.99	gi|59802419|ref|YP_209131.1|
ND	NGO0021	putative TonB-dependent receptor protein, iron related	Outer Membrane	9.99	gi|59800490|ref|YP_207202.1|
ND	NGO0510	putative phage associated protein	Outer Membrane	9.49	gi|59800948|ref|YP_207660.1|
ND	NGO0553	putative TonB-dependent receptor	Outer Membrane	9.99	gi|59800987|ref|YP_207699.1|
ND	NGO0555	hypothetical protein	Outer Membrane	9.49	gi|59800989|ref|YP_207701.1|
ND	NGO0694	hypothetical protein	Outer Membrane	9.83	gi|59801119|ref|YP_207831.1|
ND	NGO0868	OpcA	Outer Membrane	9.99	gi|59801269|ref|YP_207981.1|
ND	NGO0952	putative TonB-dependent receptor protein	Outer Membrane	9.99	gi|59801342|ref|YP_208054.1|
ND	NGO0983	Lip	Outer Membrane	9.99	gi|59801369|ref|YP_208081.1|
ND	NGO1092	putative phage associated protein	Outer Membrane	9.52	gi|59801459|ref|YP_208171.1|
ND	NGO1431	hypothetical protein	Outer Membrane	9.92	gi|59801774|ref|YP_208486.1|
ND	NGO1559	hypothetical protein	Outer Membrane	9.93	gi|59801892|ref|YP_208604.1|
ND	NGO2086	hypothetical protein	Outer Membrane	9.92	gi|59802387|ref|YP_209099.1|
ND	NGO2109	hemoglobin-haptoglobin utilization protein B	Outer Membrane	9.99	gi|59802410|ref|YP_209122.1|
ND	NGO2111	hypothetical protein	Outer Membrane	9.52	gi|59802411|ref|YP_209123.1|

a Predictions by PSORTb version 3.0.

b Up, upregulation; down, downregulation; variable, variable regulation; D, detected but not quantified in the proteomics study; and ND, not detected in the proteomics study.

The majority of the differentially expressed outer membrane proteins (11 proteins) were upregulated in biofilm organisms, including 9 proteins that were upregulated ≥2-fold (as shown in Table 1). Furthermore, as mentioned above, outer membrane opacity protein B and OpaD were two of the most highly upregulated proteins observed in the complete study. The two outer membrane proteins detected as downregulated in biofilm organisms, TbpA and FetA, were also downregulated with high fold changes as noted above (and shown in Table 2). In addition, two hypothetical outer membrane proteins whose expression changes were categorized as variable were observed. Overall, these changes in the protein composition of the gonococcal outer membrane of biofilm organisms as compared to their planktonic counterparts were among the most dramatic differences observed in our proteomics experiment.

### Key Biosynthetic Pathways Involved in the Adaptation to Biofilm Growth

The current proteomics experiment and our published study on transcriptional profiling of gonococcal biofilms [30] were run using identical continuous-flow chambers for the production of biofilm and planktonic cells. Although it is known that proteomics and transcriptional differential changes typically show little correlation, a comparison between these datasets was performed nonetheless, as one might expect some of the more critical pathways undergoing transcriptional regulation that are more widely distributed in the biofilm and with the least temporal fluctuations might also show up in the proteomic dataset.

To assess the degree to which the two datasets provided complementary information, we submitted our lists of 83 differentially expressed genes meeting a 2-fold cutoff threshold from the transcriptional profiling study [30] and 152 differentially expressed proteins meeting a 1.5-fold cutoff threshold from the present proteomics study to the KEGG website for mapping onto KEGG biosynthetic pathways. When submitted to the website, ∼44% of our combined accession numbers could be assigned to defined KEGG pathways. (For the proteomics dataset alone, the percentage of hits mapping to KEGG pathways was considerably higher, with ∼56% of the accession numbers found.) From this subset of our results that could be evaluated, we found 13 KEGG pathways involving from 5–59 accession numbers. The following KEGG pathways had 5 or more hits each: metabolic pathways (59 hits), microbial metabolism in diverse environments (25 hits), biosynthesis of secondary metabolites (23 hits), ribosome (22 hits), oxidative phosphorylation (15 hits), purine metabolism (10 hits), pyruvate metabolism (9 hits), glycolysis/gluconeogenesis (9 hits), citrate cycle (TCA cycle) (8 hits), pyrimidine metabolism (7 hits), RNA degradation (6 hits), methane metabolism (5 hits), and pentose phosphate pathway (5 hits). While a few of these pathways drew well from both the proteomic and transcriptomic datasets, we focused our attention on some of the individual pathways most represented in the proteomics dataset.

Supplementary Figure S2 shows our 9 entries that mapped onto the KEGG pathway for pyruvate metabolism. Three proteins on the pathway (derived from our proteomics experiments) that were consistently downregulated were a putative L-lactate dehydrogenase (NGO0639), pyruvate dehydrogenase subunit E1 (AceE), and acetate kinase (ACK). Three additional proteins on the pathway, phosphoenolpyruvate synthase (NGO0200), dihydrolipoamide dehydrogenase (DLDH, NGO0915), and a putative DLDH (NGO0562), showed variable expression trends. The putative and the known DLDH mapped to the same location on the KEGG pathway, although they are not highly homologous (39.0% identity). As shown, the gene for the putative DLDH was found to be upregulated in the transcriptomic experiment. The only upregulated proteins on the pathway were dihydrolipoamide acetyltransferase (NGO0564), a putative phosphotransacetylase (NGO0214), and pyruvate kinase (PykA).

From our proteomic and transcriptomic datasets, we found 8 entries that mapped onto the KEGG pathway for the TCA cycle (Supplementary Figure S3). These included both upregulated and downregulated proteins, as well as some proteins that showed variable trends. The most significant upregulated protein on this pathway was dihydrolipoamide acetyltransferase, a highly upregulated protein that was discussed above as associated with the pyruvate metabolic pathway. Likewise, the downregulated protein AceE and two proteins with variable expression patterns, DLDH and a putative DLDH, all of which were also associated with the pyruvate metabolic pathway, mapped to the TCA cycle as well. As was the case for the pyruvate metabolic pathway, the corresponding gene for the putative DLDH was the only gene in the transcriptomic dataset mapping to this pathway.

The KEGG pathway for glycolysis/gluconeogenesis was well represented in the proteomics dataset, as shown in Supplementary Figure S4. Five upregulated proteins mapped to the pathway: phosphoglucomutase (Pgm), glucokinase (Glk), glucose-6-phosphate isomerase (Pgi), pyruvate kinase (PykA), and dihydrolipoamide acetyltransferase. Two of these proteins, dihydrolipoamide acetyltransferase and PykA, were also on the pathway for pyruvate metabolism, with dihydrolipoamide acetyltransferase involved in the TCA cycle as well. In addition to these proteins, three proteins that we classified as variably regulated, phosphopyruvate hydratase (Eno), DLDH and a putative DLDH, as well as the downregulated protein AceE, also mapped to the pathway for glycolysis/gluconeogenesis. These latter three proteins were also associated with the pyruvate metabolism and TCA cycle pathways as discussed above.

Although not associated with numerous hits, the KEGG pathway for nitrogen metabolism (reduction and fixation) contained one of the most highly upregulated gene/protein pairs in this study, nitrite reductase (AniA). A second key hit from the transcriptional profiling experiment that was presumed to be associated with this pathway was nitric oxide reductase (*norB*), although its accession number is not currently recognized in the KEGG pathway database. Other noteworthy proteins in this pathway include carbonic anhydrase, which was measured as upregulated repeatedly in the proteomics experiment, and glutamate dehydrogenase, an abundant protein that was found upregulated in the soluble fraction and downregulated in the membrane fractions.

## Discussion

The goals of this study were to evaluate differential protein expression in biofilm vs. planktonic populations of *N. gonorrhoeae* and to identify biochemical pathways relevant in biofilm formation. By fractionating the proteome into three sequential extracts, we obtained good representation of both the soluble and membrane proteins fractions. Given the inherent level of heterogeneity and complexity of bacterial biofilms, we also wanted to assess how our proteomics results compared to our previously published report on transcriptional profiling of *N. gonorrhoeae* biofilms [30] and to use both datasets in the analysis of biochemical pathways. We were aware of the generally poor correlation of transcriptome and proteomics experiments but felt nonetheless that correlating these two datasets might identify some of the more robust features of biofilm formation. In the direct correlation of the two datasets, 152 proteins and 83 genes were compared with only 7 overlapping hits (Table 4). While this low number of correlations was not statistically significant, 4 of the correlations were striking in that they involved some of the most highly upregulated hits from both datasets.

**Table 4:** Correlation of differentially expressed genes and proteins in *N. gonorrhoeae* biofilms: Biofilm/Planktonic (B/P) ratios expressed as log_2_ values.

		Gene	Protein	Extract 1		Extract 2		Extract 3	
Accession	Name	B/P^a^	B/P	Avg^b^	(N)^c^	Avg^b^	(N)^c^	Avg^b^	(N)^c^
NGO1276	AniA; nitrite reductase (NO-forming)	2.434	up	3.485	(1)	3.175	(1)	1.742	(1)
NGO0070	outer membrane opacity protein B	1.507	up					2.379	(1)
NGO1769	CcpR; cytochrome c peroxidase	1.399	up	3.271	(1)	1.547	(2)		
NGO0562	putative dihydrolipoamide dehydrogenase	1.390	variable	0.996	(3)	−0.731	(3)	−1.142	(2)
NGO0340	putative cysteine synthase/cystathionine beta-synthase	1.130	up			1.378	(3)	0.816	(1)
NGO0905	hypothetical protein	−1.004	up	0.783	(2)	0.643	(1)		
NGO1494	putative ABC transporter, periplasmic binding protein, polyamine	−1.244	variable	1.287	(3)			−0.970	(1)

a Gene fold changes (RMA-filtered results from the dataset referred to in Falsetta *et al*., 2009 [30]). The fold change ratios given in the published report were converted to log_2_ values for this correlation table.

b Average log_2_ values for biofilm/planktonic protein ratios.

c (N) represents the number of measurements (biological replicates) averaged.

Using transcriptional profiling, Falsetta et al. previously showed that several genes used for anaerobic respiration were upregulated in *N. gonorrhoeae* biofilms [30]. The three key genes upregulated more than 2-fold and also validated by qRT-PCR were nitrite reductase (*aniA*), nitric oxide reductase (*norB*), and cytochrome c peroxidase (*ccp*). In the current proteomic study, we obtained independent evidence at the protein expression level that further supports the roles of nitrite reductase and cytochrome c peroxidase in biofilm growth. Indeed, AniA and CcpR were the two most highly upregulated proteins detected in our proteomics experiment (with measured fold changes in the 9- to 11-fold range) and they both were quantified repeatedly with p-values <0.05 (Table 1). In addition to being expressed under anaerobic conditions as part of the partial denitrification pathway, both of these proteins are also known to be upregulated in response to oxidative stress [44]. Mechanisms for oxidative stress tolerance appear to be required in order to sustain robust *N. gonorrhoeae* biofilms [45].

In addition to these proteins, our study also identified a number of other highly upregulated proteins involved in energy metabolism, particularly in the role of electron transport (see Table 1). Among these proteins were cbb3-type cytochrome c oxidase subunit II and cb-type cytochrome c oxidase subunit III (CcoP). The bacterial cbb3-type cytochrome c oxidases have been previously found to be expressed in response to oxygen-limited environments [46]. Furthermore, recent studies on the CcoP subunit of the gonococcal cytochrome oxidase *cbb_3_* have indicated that it plays a physiologically significant role in nitrite reduction [47]. CcoP is larger than the orthologous subunits from other bacteria and was proposed to have a third heme-binding domain that is specifically involved in electron transfer to AniA [47]. This domain was postulated to span the periplasm, thus allowing for electron transfer from cytochrome oxidase in the cytoplasmic membrane to AniA in the outer membrane [47]. Thus, the detection of CcoP as highly upregulated in the present study, along with high levels of AniA, is consistent with this new role for a cytochrome oxidase. Previously we speculated that in *N. gonorrhoeae*, there may be a complex interaction between aerobic respiration and the partial denitrification pathway [45]; the interaction between the CcoP subunit and AniA may be the link between these pathways. These data should assist in our future efforts to further define how gonococcal electron transfer pathways adjust to reduce nitrite when oxygen is limited.

There were also several additional proteins involved in energy metabolism that appeared to be differentially expressed in gonococcal biofilms. Several of these proteins mapped to three interrelated KEGG biosynthetic pathways: the pathways for glycolysis/gluconeogenesis, pyruvate metabolism, and the TCA cycle. Four upregulated proteins were enzymes involved in the conversion of glucose to pyruvate: phosphoglucomutase (Pgm), glucose-6-phosphate isomerase, glucokinase, and pyruvate kinase. In a recent study, Pgm was found to be upregulated in meningococcal biofilms as well [28]. The expression of meningococcal *pgmB* is also regulated by the global transcription activator FNR that responds to oxygen limitations [28]. Several *N. gonorrhoeae* proteins that mapped to all three of the above-listed KEGG biosynthetic pathways were involved in the conversion of pyruvate to acetyl-CoA. Dihydrolipoamide acetyltransferase was highly upregulated and measured repeatedly in the soluble fraction (Extract 1) in our proteomics experiment, whereas both a known and a putative dihydrolipoamide dehydrogenase exhibited variable expression trends in different extracts (both measured as upregulated in Extract 1 and downregulated in one or both of the other extracts). In a comparative proteomic analysis of *S. aureus* biofilm and planktonic cells, Resch et al. [25] also observed the upregulation of enzymes involved in pyruvate and formate metabolism. In addition to the three proteins mentioned above, another upregulated protein of the TCA cycle that we observed was succinate dehydrogenase iron-sulfur subunit (SdhB). It was previously reported that succinate dehydrogenase genes are upregulated in *S. aureus* biofilms [25], [26]. Taken together, these findings suggest that upregulation of sugar fermentation pathways and TCA cycle enzymes involved in catabolism and energy metabolism is advantageous under conditions where nutrients and oxygen are limiting, such as would prevail at the basal levels of bacterial biofilms [30], [45], [48].

Several proteins were identified that were upregulated in the biofilm state that are associated with the cell envelope (see Table 1). Among these were two pilus assembly proteins, PilG and PilQ, and one pilus-associated protein (NGO0055). Type IV pili have been previously associated with biofilm formation in other organisms such as *P. aeruginosa* [49], [50], *Haemophilus influenzae* [51], [52], and *Moraxella catarrhalis* [53]. The pili are thought to be important for initial attachment of cells to abiotic and biotic surfaces [54], [55], [56]. Thus, the upregulation of the *N. gonorrhoeae* proteins associated with type IV pili is likely to be associated with the initial phase of establishment and expansion of the biofilm.

Compared to planktonic organisms, we found that the protein composition of the outer membrane, as well as the cell envelope proteins mentioned above, was notably altered in gonococcal biofilms. The membrane fraction is typically not as amendable to proteomic studies as the soluble fraction, and thus has been more difficult to investigate quantitative proteomic differences in. However, recent studies have also highlighted the value of stable isotope labeling to investigate bacterial membrane proteomes [37], [39]. In our proteomics study, we detected 11 upregulated outer membrane proteins (Table 3), most of which were upregulated more than 2-fold. Outer membrane opacity protein B (OpaB) was strongly upregulated in both the proteomic and transcriptomic datasets (see Table 4). In *E. coli*, it was previously shown that the major outer membrane protein OmpA is overexpressed in biofilm cells [22]. When coupled with other physicochemical characterization techniques, quantitative proteomics helped to reveal differences in surface charge and adhesiveness of *E. coli* biofilm organisms as compared to their planktonic counterparts [21]. The bacterial outer membrane is important not only for maintaining cell integrity, but also for facilitating other processes such as signaling, energy generation, waste disposal, and regulation of nutrient supplies [37]. Clearly, bacterial outer membranes are keenly involved in the cell-cell interactions that define the biofilm lifestyle, and it is intriguing to speculate whether the upregulation of proteins known to be associated with cell adhesion and invasion, e.g., OpaB and OpaD, increase the ability of gonococcal biofilm organisms to invade host epithelial cells. Additionally, in *N. gonorrhoeae,* the biofilm extracellular matrix has been shown to contain large amounts of membranous materials arising from blebbing of the gonococcal outer membrane [5], [6]. Thus, the differentially expressed outer membrane proteins that we observed for *N. gonorrhoeae* biofilm organisms provide specific insights into how the adaptation to the biofilm growth form occurs at the cell surface. Indeed, blebbing is crucial to gonococcal biofilm formation, in part due the delivery of DNA for matrix formation [11], [12]. A number of gonococcal membrane proteins present in gonococcal blebs bind both single and double stranded DNA [57], suggesting that these membrane proteins may form a lattice over which the DNA matrix is supported. This would be similar to the observation in nontypeable *Haemophilus influenzae*, which also has a biofilm matrix containing DNA, in that DNA binding proteins in the bacterial membrane have been found to be important in maintaining biofilm integrity [58].

Along with proteins upregulated in gonococcal biofilms relative to the planktonic organisms a number of proteins were found to be significantly downregulated (>2-fold) (Table 2). Of these, the ferric enterobactin receptor (FetA), an iron complex outer membrane receptor protein, was the most highly downregulated protein. Proteins in the transport and binding class were also downregulated, including the transferrin-binding proteins (TbpA and TbpB) and the biopolymer transport protein ExbB. Both TbpA and TbpB were more than 2-fold lower in biofilm populations when compared to planktonic organisms (Table 2). In a recent microarray analysis of iron-responsive genes of *N. gonorrhoeae*, a number of genes encoding transport functions were found to be derepressed under iron-depleted growth conditions [59]. Among these were *fetA, tbpAB*, and *exbB*. The downregulation of these transport and binding proteins under biofilm growth conditions may reflect the reduced metabolic and growth rates of organisms in the biofilm state as compared to the planktonic state or may be due to a response to other environmental conditions, rather than correlate directly with iron availability. Consistent with this rationalization, it was recently observed that *S. aureus* biofilm formation is induced in iron-restricted growth conditions *in vitro*, and involves the expression of the intercellular adhesion operon *ica* [60]. While we have not investigated the growth of *N. gonorrhoeae* biofilms under iron-restricted conditions, our proteomics results clearly show that the metabolic phenotype of biofilm organisms involves altered expression of specific iron transport and binding proteins. In the human environment, gonococci scavenge iron from a number of human iron binding proteins including transferrin and lactoferrin. As neither of these proteins are present in the *in vitro* conditions used to generate the biofilm in our study, some of the changes in protein expression could reflect the lack of these natural sources of iron [61].

In contrast to these downregulated transport and binding proteins mentioned above, there were a few proteins in this same functional category that were upregulated in biofilm populations, such as bacterioferritin, a putative TonB-dependent receptor protein, and two putative ABC transporter periplasmic binding proteins (Table 1). The upregulation of these particular transport and binding proteins, and the downregulation of those discussed above, may be a reflection of the metabolic heterogeneity of bacterial biofilms. When we monitored the induction of anaerobic respiration in *N. gonorrhoeae* biofilms using a transcriptional fusion to the *aniA* gene (*aniA′-′gfp*), we observed that AniA is expressed in the lower two-thirds of the biofilm, but not at the bulk-fluid interface [48]. This suggested that cells in the substrata of gonococcal biofilms are growing anaerobically, whereas in the upper strata of the biofilm, where oxygen is more readily available, aerobic respiration is likely to predominate. Cells nearest the bulk-fluid interface are more actively metabolizing, whereas organisms in the substrata are less metabolically active. Thus, when compared to planktonic organisms, differential protein expression in mature biofilms reflects the sum total of a complex microbial community with members existing in different microenvironments.

It is also worth commenting on the experimental design we used in this study, and some of the limitations that were encountered. First, we chose to use planktonic organisms collected from the biofilm runoff, arguably the most biologically relevant control, to limit any potential artifacts associated with the growth conditions. However, it is possible that this design may have limited the differences we observed between these two states. Second, in preparing three separate extracts of increasing stringency of protein solubilization, we had hoped to increase our overall proteomic coverage, especially of less soluble membrane proteins. Indeed, our coverage of membrane proteins was relatively high: we detected ∼28% of the predicted outer membrane plus cytoplasmic membrane proteins from *N. gonorrhoeae* [62], [63]), and roughly 2/3 of the differentially expressed proteins were identified in only one of the three extracts (see Figure 4). Generally, proteins that were identified in more than one biological replicate showed consistent expression trends. However, there were ∼25 proteins that were detected in more than one of the three extracts that showed different or ‘variable’ expression trends. While there may be technical reasons for some of these mixed assignments, it is more likely that biological variations in biofilm itself were a major factor. We and other investigators, for example, have noted that these bacterial biofilms are highly heterogeneous, and despite attempts to make these replicates as reproducible as possible in this current study, each experiment has its own temporal and spatial outcome [48], [64]. It is also possible that some of these proteins whose expression trends were variable among the three extracts could reflect their translocation to other cellular compartments with altered solubility characteristics. Third, for mostly technical reasons, we were restricted to a single time point before harvesting each biofilm culture in its entirety. Ideally, one would like to follow multiple time points as well as assessing differences in the spatial distribution of proteins within the biofilms. Given the combination of transcriptomic and now proteomic data that is available for gonococcal biofilms, it is now worth considering targeting sets of proteins and networks using immunological staining techniques, if the antibodies are available. Fourth, it is also possible that changes in the posttranslational modification status of some proteins might accompany biofilm formation as well. Although we did not exclude such possibilities in our analysis of the proteomic data, modifications such as phosphorylation – which are known to occur in *Neisseria* [65] and other bacterial species – typically require an affinity enrichment step to be observed in complex mixtures. And lastly, an early technical decision to run 2D gels in parallel with the 1D SDS-PAGE-based proteomic approach may have added to technical variation in our proteomic measurements. While this approach did provide visual confidence early on that useful and important protein differences between the planktonic and biofilm populations could be observed, we likely would have obtained more technical reproducibility by mixing the two populations at the start – prior to making the three extracts. Indeed, we have recently carried out such a SILAC experiment to study changes in *Haemophilus influenzae* biofilms (unpublished data) using a 1D gel approach only (final data analysis in progress).

In summary, we used quantitative SILAC labeling to compare planktonic to biofilm organisms in *N. gonorrhoeae*, and a relatively large set of 152 proteins were found to be differentially expressed. We believe it is critical that proteomic experiments be carried out in addition to transcriptomic studies, as there are numerous studies showing large discrepancies between these two approaches. In recent years, proteomics techniques have been used to compare several species of bacteria in biofilm vs. planktonic growth modes, including *P. aeruginosa* [19], *S. aureus* [25], *Acinetobacter baumannii* [66], *Escherichia coli* [21], [22] and *N. meningitidis* [28]. However, to our knowledge, none of these proteomic studies used SILAC or provided the depth of discovery as seen here for gonococcal biofilms. In addition, we previously showed by transcriptional profiling that the metabolic phenotype of gonococcal biofilms is similar whether the organism is grown over glass (in our continuous-flow chamber) or grown over transformed human cervical epithelial cells [30], [45]. Consequently, the protein expression changes associated with *N. gonorrhoeae* biofilms observed in this study are likely to provide a reliable view of the metabolic state of the organism in naturally occurring biofilms. However, one unavoidable limitation of our strategy was the sampling of the highly heterogeneous biofilm structure, thus providing an ‘average’ picture of the proteins expressed in these samples. Therefore, the differential protein expression of the planktonic bacteria versus the biofilm organisms from various microenvironments of the biofilm may be even larger than we observed in our current study. As an extension of our previous transcriptomic analysis [30], our proteomics survey further demonstrates the role of anaerobic respiration in the biofilm growth mode and highlights the metabolic heterogeneity of gonococcal biofilms. However, by directly examining protein expression changes in these biofilms, the proteomic data clearly demonstrated the importance of energy metabolism in biofilms as well as expanded our understanding of electron transport pathways in *N. gonorrhoeae*. In addition, preliminary studies suggest that quorum sensing may also play a role in gene regulation and protein expression during biofilm formation and dispersal, and experiments are currently underway to investigate this observation and its impact on both planktonic and biofilm organisms (unpublished data). In conclusion, these proteomic data could provide a set of targets for future immunological staining studies, providing spatial resolution data of biofilm organization that was not possible in this current study. Furthermore, 23 of 37 (62%) of the predicted gonococcal outer membrane proteins were detected [62], [63], and 15 of these were differentially expressed in biofilms. These biofilm-enriched outer membrane proteins may offer a particularly attractive target set for reagent development for rapid and accurate diagnostic modalities.

## Supporting Information

Figure S1 2D SDS-PAGE gels of soluble proteins (Extract 1) from (A) planktonic organisms, (B) biofilm organisms, and (C) planktonic + biofilm organisms. Circled protein spots in panels A and B show visible intensity differences between the two gels. Fifty-five spots were excised from the mixed gel in panel C as indicated.
(PDF)

Figure S2 KEGG pathway for pyruvate metabolism. Differentially expressed *N. gonorrhoeae* proteins from the present study and genes from the published transcriptional profiling study [30] that mapped to this KEGG pathway are color-coded on the pathway and listed in the Key.
(PDF)

Figure S3 KEGG pathway for the citrate cycle (TCA cycle). Differentially expressed *N. gonorrhoeae* proteins from the present study and genes from the published transcriptional profiling study [30] that mapped to this KEGG pathway are color-coded on the pathway and listed in the Key.
(PDF)

Figure S4 KEGG pathway glycolysis/gluconeogenesis. Differentially expressed *N. gonorrhoeae* proteins from the present study and genes from the published transcriptional profiling study [30] that mapped to this KEGG pathway are color-coded on the pathway and listed in the Key.
(PDF)

Table S1 Differentially expressed proteins detected in the pilot study using 2D SDS-PAGE for analysis of soluble proteins from mixed Extract 1.
(PDF)

Table S2 Excel workbook with 5 sheets listing proteins and their associated peptides. **Sheet 1:** Sorted table listing all of the quantified proteins with p-values <0.05. Columns contain results for individual datasets. **Sheet 2:** Peptides used in the quantification of the proteins with p-values <0.05 given in Sheet 1. **Sheet 3:** Sorted table listing all of the proteins identified and quantified in the full proteomics study (all proteins with Unused ProtScores of ≥2.0). Proteins that were detected but not quantified are marked with an ‘x’. L:H ratios for quantified proteins with p-values <0.05 are given in black type. L:H ratio entries in red type represent quantified proteins with poor statistics (p-values >0.05). **Sheet 4:** Peptides used in the identification and quantification of the proteins are listed under each entry. Only peptides with a confidence score of ≥50% are included in the table. The peptide level results are color-coded as follows: black for quantified peptides used in the quantification, red for quantified peptides not used in the quantification, and ‘x’ for peptides contributing to the identification only. **Sheet 5:** Notes pertaining to the 4 datasheets.
(XLS)

Table S3 Excel spreadsheet listing only the differentially expressed proteins with p-values <0.05 observed in the full proteomics study. This table shows all of the raw coverage and statistical information for each protein entry.
(XLS)

Table S4 Excel workbook with 3 sheets showing complete peptide level information for all of the quantified proteins (p-values <0.05) from Extract 1 (3 biological replicates). Only peptides with a confidence score of ≥50% are included in the table. **S4B.** Excel workbook with 3 sheets showing complete peptide level information for all of the quantified proteins (p-values <0.05) from Extract 2 (3 biological replicates). Only peptides with a confidence score of ≥50% are included in the table. **S4C.** Excel workbook with 3 sheets showing complete peptide level information for all of the quantified proteins (p-values <0.05) from Extract 3 (3 biological replicates). Only peptides with a confidence score of ≥50% are included in the table.
(ZIP)

Table S5 Average values (log_2_) for all of the upregulated proteins detected in the study with p-values <0.05 and meeting a 1.5-fold cutoff threshold. ‘N’ indicates the number of measurements averaged in each Extract. Functional classifications for the proteins were downloaded from the JCVI-CMR website (http://cmr.jcvi.org/tigr-scripts/CMR/CmrHomePage.cgi).
(PDF)

Table S6 Average values (log_2_) for all of the downregulated proteins detected in the study with p-values <0.05 and meeting a 1.5-fold cutoff threshold. ‘N’ indicates the number of measurements averaged in each Extract. Functional classifications for the proteins were downloaded from the JCVI-CMR website (http://cmr.jcvi.org/tigr-scripts/CMR/CmrHomePage.cgi).
(PDF)

Table S7 Average values (log_2_) for all of the quantified proteins detected in the study with p-values <0.05 and meeting a 1.5-fold cutoff threshold whose expression trends varied in different Extracts. ‘N’ indicates the number of measurements averaged. Functional classifications for the proteins were downloaded from the JCVI-CMR website (http://cmr.jcvi.org/tigr-scripts/CMR/CmrHomePage.cgi).
(PDF)
