# Supplementary material for: Proteomic Analysis of Neisseria gonorrhoeae Biofilms Shows Shift to Anaerobic Respiration and Changes in Nutrient Transport and Outermembrane Proteins
Source: PLoS One. 2012 Jun 6;7(6):e38303. doi: 10.1371/journal.pone.0038303 (PMC3368942; doi:10.1371/journal.pone.0038303)
Supplement: Figure S1 — 2D SDS-PAGE gels of soluble proteins (Extract 1) from (A) planktonic organisms, (B) biofilm organisms, and (C) planktonic + biofilm organisms. Circled protein spots in panels A and B show visible intensity differences between the two gels. Fifty-five spots were excised from the mixed gel in panel C as indicated. (PDF) [file pone.0038303.s001.pdf]

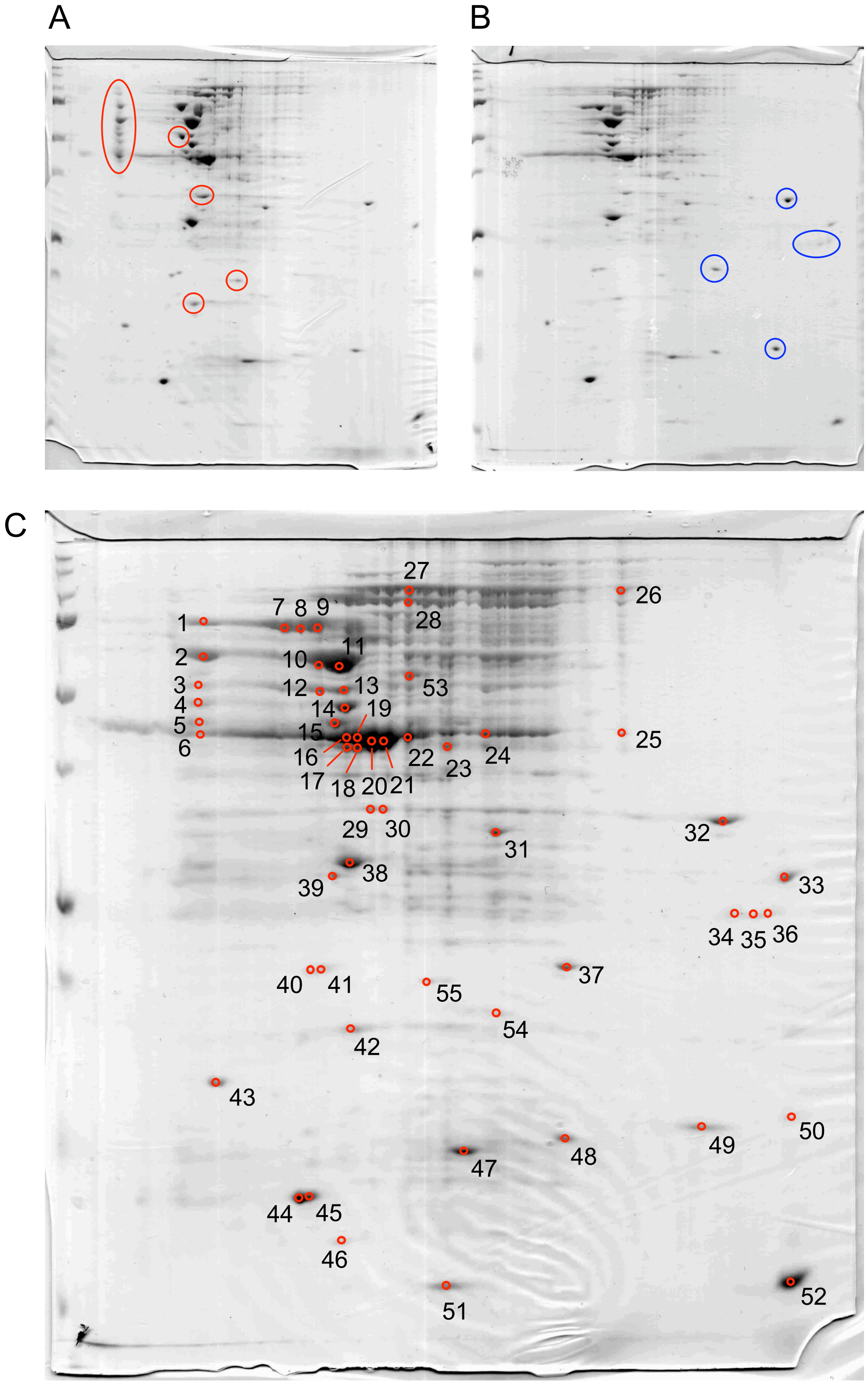

Supplementary Figure S1. 2D SDS-PAGE gels of soluble proteins (Extract 1) from (A) planktonic organisms, (B) biofilm organisms, and (C) planktonic + biofilm organisms. Circled protein spots in panels A and B show visible intensity differences between the two gels. Fifty five spots were excised from the mixed gel in panel C as indicated.
