# Supplementary figures and images for: Proteomic Analysis of Neisseria gonorrhoeae Biofilms Shows Shift to Anaerobic Respiration and Changes in Nutrient Transport and Outermembrane Proteins
Source: PLoS One. 2012 Jun 6;7(6):e38303. doi: 10.1371/journal.pone.0038303 (PMC3368942; doi:10.1371/journal.pone.0038303)

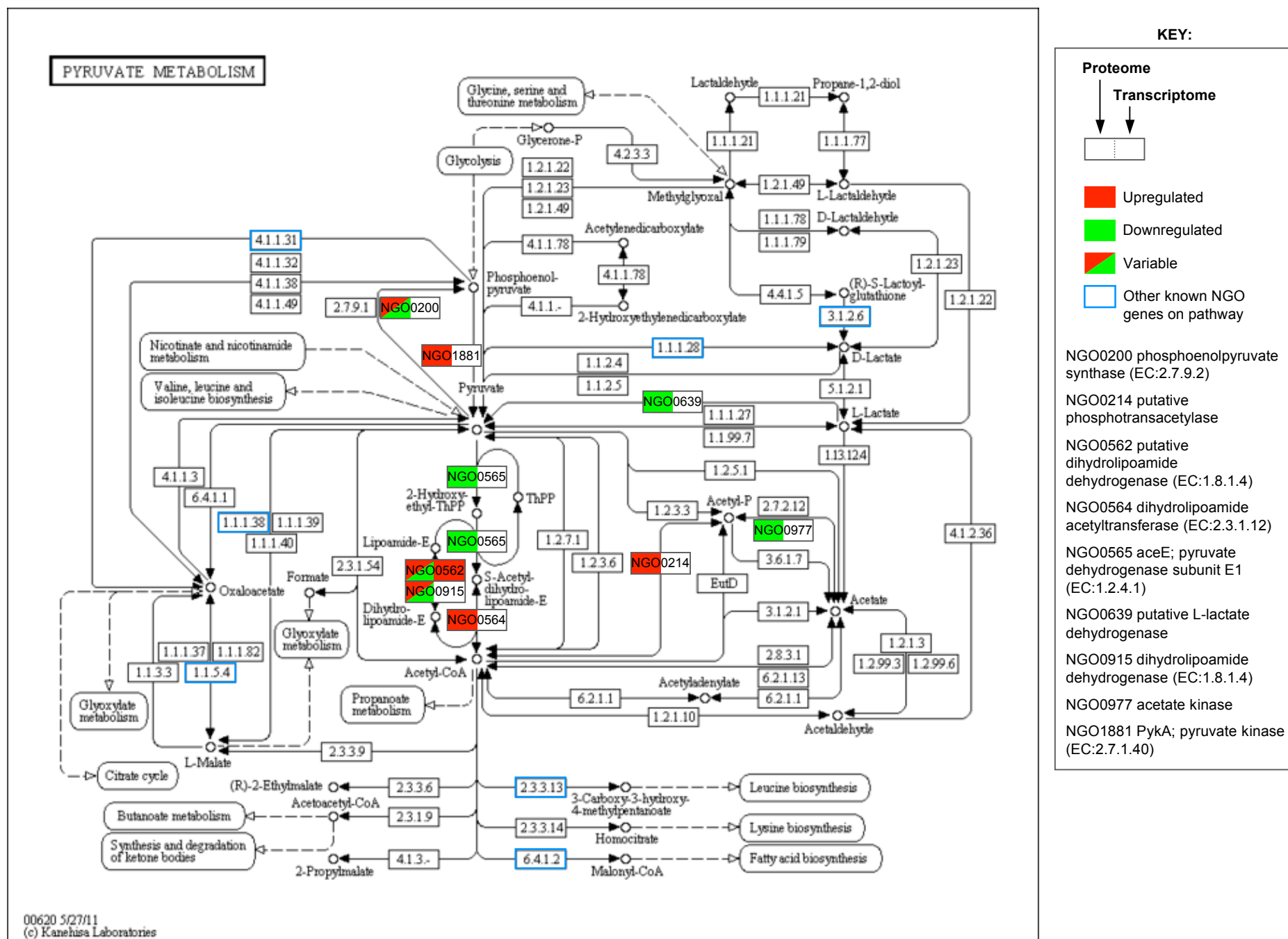

Supplementary Figure S2. KEGG pathway for pyruvate metabolism.

Supplement: Figure S2 — KEGG pathway for pyruvate metabolism. Differentially expressed N. gonorrhoeae proteins from the present study and genes from the published transcriptional profiling study [30] that mapped to this KEGG pathway are color-coded on the pathway and listed in the Key. (PDF) [file pone.0038303.s002.pdf]

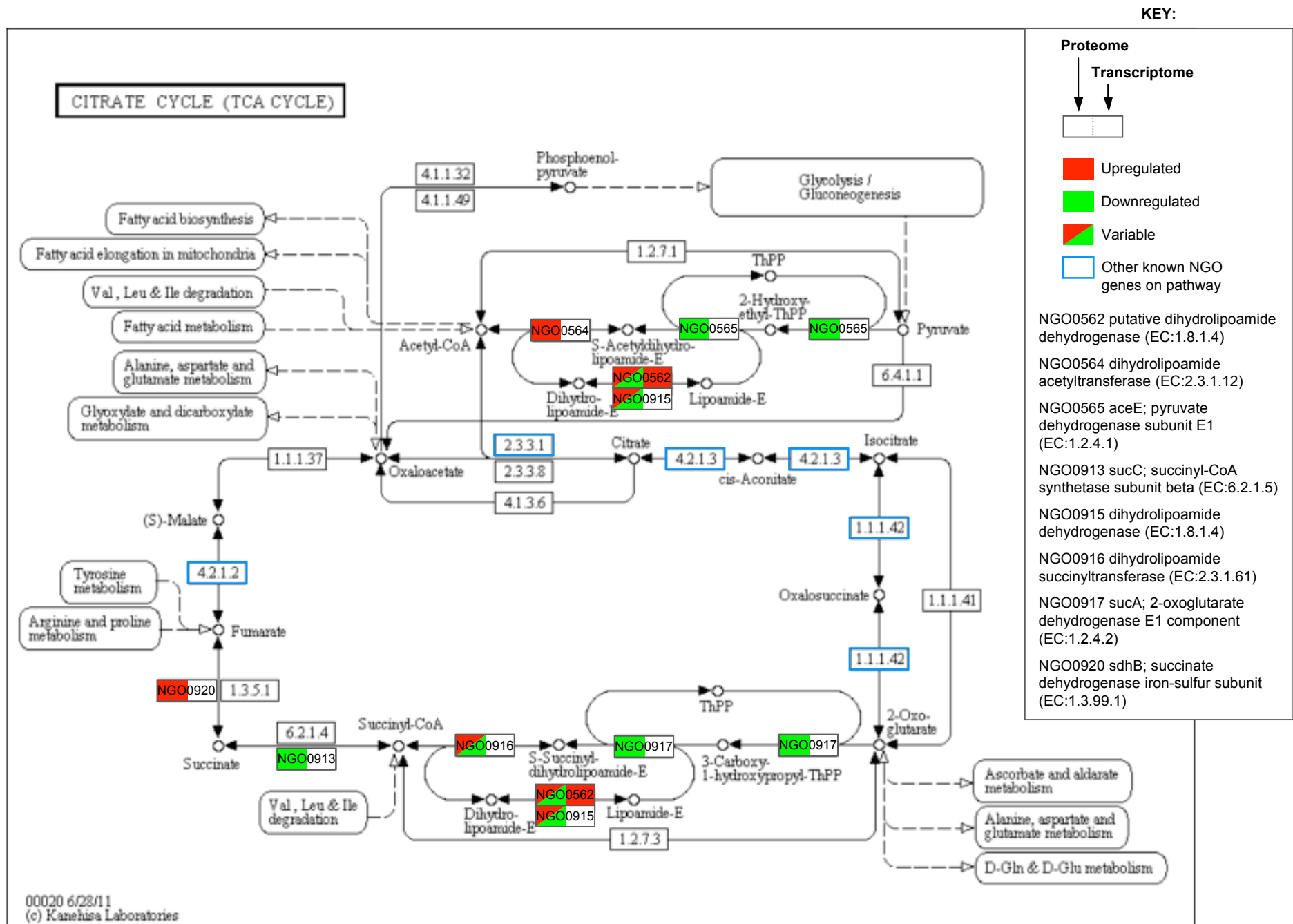

Supplementary Figure S3. KEGG pathway for the citrate cycle (TCA cycle).

Supplement: Figure S3 — KEGG pathway for the citrate cycle (TCA cycle). Differentially expressed N. gonorrhoeae proteins from the present study and genes from the published transcriptional profiling study [30] that mapped to this KEGG pathway are color-coded on the pathway and listed in the Key. (PDF) [file pone.0038303.s003.pdf]

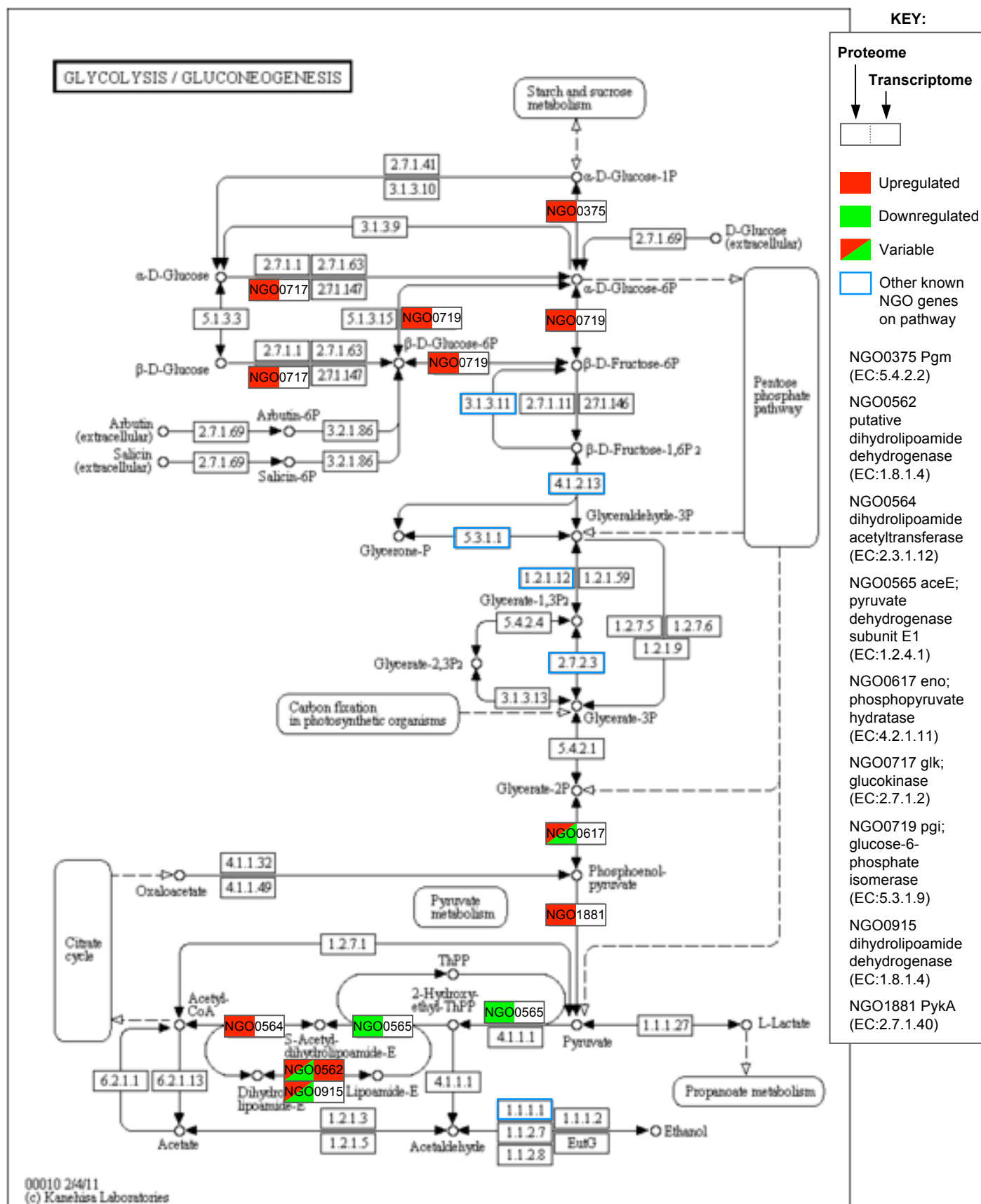

Supplementary Figure S4. KEGG pathway for glycolysis/gluconeogenesis.

Supplement: Figure S4 — KEGG pathway glycolysis/gluconeogenesis. Differentially expressed N. gonorrhoeae proteins from the present study and genes from the published transcriptional profiling study [30] that mapped to this KEGG pathway are color-coded on the pathway and listed in the Key. (PDF) [file pone.0038303.s004.pdf]
