# Supplementary material for: Proteomic Analysis of Neisseria gonorrhoeae Biofilms Shows Shift to Anaerobic Respiration and Changes in Nutrient Transport and Outermembrane Proteins
Source: PLoS One. 2012 Jun 6;7(6):e38303. doi: 10.1371/journal.pone.0038303 (PMC3368942; doi:10.1371/journal.pone.0038303)
Supplement: Table S1 — Differentially expressed proteins detected in the pilot study using 2D SDS-PAGE for analysis of soluble proteins from mixed Extract 1. (PDF) [file pone.0038303.s005.pdf]

Supplementary Table S1. Differentially expressed proteins detected in the preliminary 2D SDS-PAGE study, all with p-values <0.05.[a]

gel spot(s)	Accession	Name	# peptides (95% conf)	L:H[b]	$\log_2$ L:H[c]	p-value L:H
**Upregulated in biofilm**						
34[d]	NGO0574	Cah; carbonic anhydrase	14	5.203	2.379	0.03179
32	NGO1812	major outer membrane protein porin P.IB	25	1.871	0.904	0.00065
7	NGO0562	putative dihydrolipoamide dehydrogenase	11	1.526	0.610	0.00082
48	NGO18311	RplP; 50S ribosomal protein L16	3	1.385	0.470	0.00404
18, 19, 20	NGO0916	dihydrolipoamide succinyltransferase	6	1.352	0.435	0.00359
5, 15	NGO0617	Eno; phosphopyruvate hydratase	34	1.341	0.424	0.00985
47	NGO0171	RplS; 50S ribosomal protein L19	1	1.261	0.335	0.04409
23	NGO1919	Pgk; phosphoglycerate kinase	36	1.227	0.295	0.00263
**Downregulated in biofilm**						
55	NGO0108	hypothetical protein	14	0.512	-0.965	0.03702
42	NGO1871	Def; peptide deformylase	6	0.578	-0.790	0.00531
42	NGO0376	putative peptidyl-prolyl cis-trans isomerase B	16	0.703	-0.508	0.01966
47	NGO2094	GroES; co-chaperonin GroES	18	0.733	-0.449	4.79E-05
18, 23	NGO0913	SucC; succinyl-CoA synthetase subunit beta	30	0.750	-0.414	0.03648
22	NGO0977	acetate kinase	19	0.807	-0.310	0.00077
23	NGO1901	chaperone protein DnaJ	4	0.900	-0.152	0.04698

[a] In this preliminary study, MS files were searched and quantified using ProteinPilot 3.0 (revision 114732) running the Paragon Algorithm 3.0.0.0, 113442.
[b] Raw light to heavy (L:H) ratios reflecting biofilm to planktonic protein ratios.
[c] L:H ratios converted to $\log_2$ values.
[d] Carbonic anhydrase was also detected in gel spots 35 and 36 by MALDI-TOF MS.
