# Supplementary material for: Proteomic Analysis of Neisseria gonorrhoeae Biofilms Shows Shift to Anaerobic Respiration and Changes in Nutrient Transport and Outermembrane Proteins
Source: PLoS One. 2012 Jun 6;7(6):e38303. doi: 10.1371/journal.pone.0038303 (PMC3368942; doi:10.1371/journal.pone.0038303)
Supplement: Table S5 — Average values (log2) for all of the upregulated proteins detected in the study with p-values <0.05 and meeting a 1.5-fold cutoff threshold. ‘N’ indicates the number of measurements averaged in each Extract. Functional classifications for the proteins were downloaded from the JCVI-CMR website (http://cmr.jcvi.org/tigr-scripts/CMR/CmrHomePage.cgi). (PDF) [file pone.0038303.s009.pdf]

Supplementary Table S5. Upregulated proteins in *N. gonorrhoeae* biofilms listed by functional roles[a]: Biofilm/Planktonic ratios expressed as $\log_2$ values, 1.5-fold cutoff threshold ($\log_2$ values ≥ 0.585), p-values <0.05)

Accession	Name	JCVI sub-role	Extract 1 Avg[b]	(N)[c]	Extract 2 Avg[b]	(N)[c]	Extract 3 Avg[b]	(N)[c]
**Amino acid biosynthesis**								
NGO1242	HisB; imidazoleglycerol-phosphate dehydratase	Histidine family	1.331	(1)				
NGO0340	putative cysteine synthase/cystathionine beta-synthase	Serine family			1.378	(3)	0.816	(1)
NGO0674	3-isopropylmalate dehydrogenase	Pyruvate family			0.674	(2)		
**Biosynthesis of cofactors, prosthetic groups, and carriers**								
NGO1217	glutathione synthetase	Glutathione and analogs			0.959	(2)		
**Cell envelope**								
NGO1513	OpaD	Other	2.018	(2)	1.552	(2)	2.549	(2)
NGO0055	pilus-associated protein	Surface structures	0.806	(2)	1.324	(1)		
NGO1577	Omp3	Other	0.656	(3)			0.857	(3)
NGO1669	PilG; type IV pilus assembly protein PilC	Surface structures			1.276	(1)	0.644	(2)
NGO0233	outer membrane protein	Other			1.038	(2)	1.093	(3)
NGO1873[e]	hypothetical protein	Biosynthesis and degradation of surface polysaccharides and lipopolysaccharides			0.757	(3)	0.870	(3)
NGO1802	hypothetical protein; outer membrane protein	Other			0.665	(1)		
NGO0070	outer membrane opacity protein B	Other					2.379	(1)
NGO1949[f]	hypothetical protein	Other					1.739	(1)
NGO0948[g]	hypothetical protein; lipoprotein-34	Other					1.222	(3)
NGO1801	hypothetical protein	Other					1.199	(1)
NGO0094[d]	hypothetical protein; type IV pilus assembly protein PilQ	Surface structures					1.098	(3)
NGO1251[h]	hypothetical protein	Other					1.045	(1)
**Cellular processes**								
NGO1225	putative peptidyl-prolylisomerase	Pathogenesis	1.387	(1)				
NGO1767	KatA; catalase	Detoxification	0.954	(3)				
NGO1382	putative GTP pyrophosphokinase	Adaptations to atypical conditions			1.348	(1)		
NGO1363[d]	hypothetical protein	Detoxification			0.643	(1)	0.774	(2)
NGO0382	hypothetical protein; cell division protease FtsH	Cell division					1.071	(1)
NGO0277	ComL; putative lipoprotein	DNA transformation					1.031	(2)
NGO2119[d]	hypothetical protein; putative toluene tolerance protein	Toxin production and resistance					0.743	(1)
**Central intermediary metabolism**								
NGO1276	AniA; nitrite reductase (NO-forming)	Nitrogen metabolism	3.485	(1)	3.175	(1)	1.742	(1)
NGO0574	Cah; carbonic anhydrase	Other	1.525	(3)	0.668	(2)		
**DNA metabolism**								
NGO1031	putative single-stranded DNA binding protein	DNA replication, recombination, and repair	0.778	(1)				
NGO1863	DNA topoisomerase I	DNA replication, recombination, and repair	0.621	(1)				
NGO0423	RdgC; recombination associated protein	DNA replication, recombination, and repair	0.617	(1)				
**Energy metabolism**								
NGO1769	CcpR; cytochrome c peroxidase	Electron transport	3.271	(1)	1.547	(2)		
NGO0564	dihydrolipoamide acetyltransferase	Pyruvate dehydrogenase	1.724	(3)				
NGO1812[d]	major outer membrane protein porin P.IB	Electron transport	1.669	(3)	0.908	(3)	0.697	(3)
NGO2146	F0F1 ATP synthase subunit B; F-type H+-transporting ATPase subunit b	ATP-proton motive force interconversion	1.637	(2)	0.717	(2)	0.593	(3)
NGO0906	hypothetical protein	Electron transport	1.073	(1)	0.614	(1)		
NGO0717	Glk; glucokinase	Glycolysis/gluconeogenesis	0.781	(3)				
NGO0719	Pgi; glucose-6-phosphate isomerase	Glycolysis/gluconeogenesis	0.765	(2)				
NGO1881	PykA; pyruvate kinase	Glycolysis/gluconeogenesis	0.644	(1)				
NGO0920	SdhB; succinate dehydrogenase iron-sulfur subunit	TCA cycle	0.609	(1)				
NGO1373	cbb3-type cytochrome c oxidase subunit II	Electron transport			1.479	(1)	1.930	(1)
NGO0375	Pgm; phosphoglucomutase	Sugars			1.368	(1)		
NGO0718[d]	RpiR family transcriptional regulator	Glycolysis/gluconeogenesis			1.217	(1)		
NGO0214	putative phosphotransacetylase	Fermentation			1.177	(2)		
NGO1328	putative cytochrome	Electron transport			1.152	(1)		
NGO1610	transaldolase	Pentose phosphate pathway			0.718	(1)		
NGO1080	putative C-type cytochrome	Electron transport			0.627	(1)		

NGO1371	CcoP; cb-type cytochrome c oxidase subunit III	Electron transport			1.602 (3)
NGO1985[i]	hypothetical protein	Electron transport			1.351 (3)
NGO1470	PntA; NAD(P) transhydrogenase subunit alpha	Electron transport			1.293 (1)
NGO1584[j]	MafA3; putative adhesin	Electron transport			1.255 (2)
NGO2031	PetC; ubiquinol-cytochrome c reductase cytochrome c1 subunit	Electron transport			1.233 (3)
NGO1972[k]	MafA4; putative adhesin	Electron transport			0.773 (2)
**Fatty acid and phospholipid metabolism**					
NGO1206[l]	phosphatidylserine decarboxylase	Biosynthesis			0.713 (2)
**Mobile and extrachromosomal element functions**					
NGO0523	putative phage associated protein	Prophage functions			1.161 (1)
**Protein fate**					
NGO0845	hypothetical protein	Degradation of proteins, peptides, and glycopeptides	0.957 (2)		
NGO1901	chaperone protein DnaJ	Protein folding and stabilization	0.734 (1)		
NGO0399	heat shock protein HtpX	Protein folding and stabilization		1.668 (1)	1.683 (1)
NGO1081	alpha-2,3-sialyltransferase	Protein modification and repair			0.668 (1)
**Protein synthesis**					
NGO1844	30S ribosomal protein S7	Ribosomal proteins: synthesis and modification	1.219 (1)		
NGO1832	RpsC; 30S ribosomal protein S3	Ribosomal proteins: synthesis and modification		1.325 (3)	
NGO1845	RpsL; 30S ribosomal protein S12	Ribosomal proteins: synthesis and modification		0.893 (1)	
NGO1858	Tuf; elongation factor Tu	Translation factors		0.801 (2)	
NGO1830[m]	RpsQ; 30S ribosomal protein S17	Ribosomal proteins: synthesis and modification			1.120 (3)
NGO1826[n]	RpsH; 30S ribosomal protein S8	Ribosomal proteins: synthesis and modification			0.904 (2)
**Regulatory functions**					
NGO0326[o]	hypothetical protein; host factor-I protein	Other	0.631 (2)		
**Transcription**					
NGO1285	NusA; transcription elongation factor NusA	Transcription factors		0.804 (1)	
**Transport and binding proteins**					
NGO0206	putative ABC transporter, periplasmic binding protein, polyamine	Amino acids, peptides and amines	1.217 (1)		
NGO0372	putative ABC transporter, periplasmic binding protein, amino acid	Cations and iron carrying compounds	1.109 (1)		0.609 (1)
NGO0794	BfrA; bacterioferritin	Cations and iron carrying compounds	1.090 (2)		
NGO2139	hypothetical protein; D-methionine transport system substrate-binding protein	Carbohydrates, organic alcohols, and acids	0.693 (1)		
NGO0455	hypothetical protein; type IV pilus assembly protein PilX	Unknown substrate			1.784 (1)
NGO1205	putative TonB-dependent receptor protein; iron complex outermembrane receptor protein	Cations and iron carrying compounds			1.530 (2)
**Hypothetical proteins**					
NGO0905	hypothetical protein	Conserved	0.783 (2)	0.643 (1)	
NGO0236	hypothetical protein	Conserved			1.344 (1)

[a] Functional role assignments (both main and JCVI sub-roles) for the *N. gonorrhoeae* proteins were downloaded from the JCVI-CMR website (http://cmr.jcvi.org/tigr-scripts/CMR/CmrHomePage.cgi).

[b] Average $\log_2$ values for biofilm/planktonic protein ratios. Values given in red meet the 2-fold cutoff threshold ($\log_2$ values ≥ 1.000).

[c] (N) represents the number of measurements (biological replicates) averaged.

[d] Proteins with more than one functional role assignment provided (alternate Main role/JCVI sub-role): NGO0094, Cellular processes/DNA transformation, Cellular processes/Pathogenesis; NGO1363, Transport and binding proteins/Other; NGO2119, Cellular processes/Detoxification; NGO1812, Energy metabolism/Fermentation; NGO0718, Energy metabolism/Sugars.

[e] NGO1873 tentatively reclassified based on orthology to *N. meningitidis* NMB0109 (90.4% identity), NMC0101 (89.8% identity), and NMCC_2040 (89.3% identity).

[f] NGO1949 tentatively reclassified based on orthology to *N. meningitidis* NMC2118 (96.0% identity) and NMCC_2098 (96.0% identity).

[g] NGO0948 tentatively reclassified based on orthology to *N. meningitidis* NMB0928 (97.2% identity), NMC0906 (96.5% identity), NMCC_0871 (97.3% identity), and NMA1123 (97.3% identity).

[h] NGO1251 tentatively reclassified based on orthology to *N. meningitidis* NMB1592 (95.7% identity).

[i] NGO1985 tentatively reclassified based on orthology to *N. meningitidis* NMA0339 (97.5% identity). Possible alternate Main role/JCVI sub-role assignments for this protein include: Cell envelope/Other (NMCC_2056, 95.5% identity; and NMC2071, 95.5% identity) and Cellular processes/Toxin production and resistance (NMB2091, 95.5% identity).

[j] NGO1584 tentatively reclassified based on orthology to *N. meningitidis* NMA2112 (98.4% identity). Possible alternate Main role/JCVI sub-role assignments for this protein include: Regulatory functions/Other (NMA2112), Protein Synthesis/Ribosomal proteins: synthesis and modification (NMA2112), Cell envelope/Surface structures (NMC0596, 97.8% identity), and Cellular processes/Pathogenesis (NMC0596, 97.8% identity).

[k] NGO1972 tentatively reclassified based on orthology to *N. meningitidis* NMA0325 (98.8% identity). Possible alternate Main role/JCVI sub-role assignments for this protein include: Regulatory functions/Other (NMA0325), Protein Synthesis/Ribosomal proteins: synthesis and modification (NMA0325), and Cellular processes/Pathogenesis (NMC2083, 98.4% identity).

[l] NGO1206 tentatively reclassified based on orthology to *N. meningitidis* NMA1160 (93.2% identity).

[m] NGO1830 tentatively reclassified based on orthology to *N. meningitidis* NMCC_1996 (98.9% identity), NMC0141 (98.9% identity), and NMB0151 (98.9% identity).

[n] NGO1826 tentatively reclassified based on orthology to *N. meningitidis* NMC0146 (100% identity) and NMB0156 (100% identity).

[o] NGO0326 tentatively reclassified based on orthology to *N. meningitidis* NMB0784 (97.9% identity). Possible alternate Main role/JCVI sub-role assignments for this protein include: Mobile and extrachromosomal element functions/Prophage functions (NMB0784).
