# Supplementary material for: Proteomic Analysis of Neisseria gonorrhoeae Biofilms Shows Shift to Anaerobic Respiration and Changes in Nutrient Transport and Outermembrane Proteins
Source: PLoS One. 2012 Jun 6;7(6):e38303. doi: 10.1371/journal.pone.0038303 (PMC3368942; doi:10.1371/journal.pone.0038303)
Supplement: Table S6 — Average values (log2) for all of the downregulated proteins detected in the study with p-values <0.05 and meeting a 1.5-fold cutoff threshold. ‘N’ indicates the number of measurements averaged in each Extract. Functional classifications for the proteins were downloaded from the JCVI-CMR website (http://cmr.jcvi.org/tigr-scripts/CMR/CmrHomePage.cgi). (PDF) [file pone.0038303.s010.pdf]

Supplementary Table S6. Downregulated proteins in *N. gonorrhoeae* biofilms listed by functional roles[a]: Biofilm/Planktonic ratios expressed as $\log_2$ values, 1.5-fold cutoff threshold ($\log_2$ values ≤ -0.585), p-values <0.05)

Accession	Name	JCVI sub-role	Extract 1 Avg[b]	(N)[c]	Extract 2 Avg[b]	(N)[c]	Extract 3 Avg[b]	(N)[c]
**Amino acid biosynthesis**								
NGO0212	1-(5-phosphoribosyl)-5-[(5-phosphoribosylamino)methylideneamino] imidazole-4-carboxamide isomerase	Histidine family	-0.623	(1)				
NGO0947	DapA; dihydrodipicolinate synthase	Aspartate family			-1.901	(1)		
NGO1961	argininosuccinate synthase	Glutamate family			-1.643	(1)		
NGO0397	HisZ; ATP phosphoribosyltransferase regulatory subunit	Histidine family			-1.320	(1)		
**Biosynthesis of cofactors, prosthetic groups, and carriers**								
NGO0704	bifunctional 3,4-dihydroxy-2-butanone 4-phosphate synthase/GTP cyclohydrolase II-like protein	Riboflavin, FMN, and FAD	-1.100	(1)				
NGO1684	7-cyano-7-deazaguanine reductase	Folic acid			-1.210	(1)		
**Cell envelope**								
NGO0403	ADP-L-glycero-D-mannoheptose epimerase	Biosynthesis and degradation of surface polysaccharides and lipopolysaccharides			-0.986	(1)		
NGO0346[d]	putative twitching motility-like protein; twitching motility protein PilU	Surface structures			-0.931	(2)		
**Cellular processes**								
NGO1909	putative twitching motility - like protein; twitching motility protein PilU	Chemotaxis and motility	-0.763	(1)				
NGO1814	cell division topological specificity factor MinE	Cell division	-0.586	(2)				
NGO1528	cell division protein FtsZ	Cell division					-0.951	(2)
**Energy metabolism**								
NGO0108[e]	hypothetical protein	Electron transport	-0.942	(1)				
NGO0687	putative ferredoxin-NADP reductase	Electron transport	-0.717	(1)				
NGO2147	F0F1 ATP synthase subunit delta; F-type H+-transporting ATPase subunit delta	ATP-proton motive force interconversion	-0.635	(1)				
NGO2149	F0F1 ATP synthase subunit gamma; F-type H+-transporting ATPase subunit gamma	ATP-proton motive force interconversion			-2.138	(1)		
NGO2148	F0F1 ATP synthase subunit alpha; F-type H+-transporting ATPase subunit alpha	ATP-proton motive force interconversion			-1.392	(3)	-1.621	(1)
NGO0639[d]	putative L-lactate dehydrogenase; L-lactate dehydrogenase (cytochrome)	Anaerobic			-1.096	(3)	-0.849	(3)
NGO0565	AceE; pyruvate dehydrogenase subunit E1	Pyruvate dehydrogenase			-0.875	(3)	-1.178	(3)
NGO0913	SucC; succinyl-CoA synthetase subunit beta	TCA cycle			-0.767	(1)		
NGO0977	acetate kinase	Fermentation			-0.689	(1)		
NGO0917	SucA; 2-oxoglutarate dehydrogenase E1 component	TCA cycle			-0.652	(1)		
**Protein fate**								
NGO1422	heat shock protein GrpE; molecular chaperone GrpE	Protein folding and stabilization			-2.206	(1)		
NGO1046	putative ClpB protein; ATP-dependent Clp protease ATP-binding subunit ClpB	Degradation of proteins, peptides, and glycopeptides			-1.798	(1)		
NGO0116	preprotein translocase subunit SecB	Protein and peptide secretion and trafficking			-1.572	(1)		
NGO2095	GroEL; chaperonin GroEL	Protein folding and stabilization			-1.528	(3)	-1.875	(2)
NGO1429	DnaK; molecular chaperone DnaK	Protein folding and stabilization			-0.793	(3)	-0.848	(1)
**Protein synthesis**								
NGO0066	putative tyrosyl-tRNA synthase	tRNA aminoacylation	-0.764	(1)				
NGO1841	RpsJ, nusE; 30S ribosomal protein S10	Ribosomal proteins: synthesis and modification	-0.662	(2)				
NGO0174	RpsP; 30S ribosomal protein S16	Ribosomal proteins: synthesis and modification			-1.579	(1)		
NGO0304	PheT; phenylalanyl-tRNA synthetase subunit beta	tRNA aminoacylation			-1.434	(1)		
NGO1843	FusA; elongation factor G	Translation factors			-1.381	(1)		
NGO1838	RplC; 50S ribosomal protein L3	Ribosomal proteins: synthesis and modification			-1.297	(2)		
NGO2024	RplM; 50S ribosomal protein L13	Ribosomal proteins: synthesis and modification			-1.123	(1)		
NGO0584	RplI; 50S ribosomal protein L9	Ribosomal proteins: synthesis and modification			-0.841	(3)		

NGO1854	RplA; 50S ribosomal protein L1	Ribosomal proteins: synthesis and modification		-0.731 (2)	
NGO0295	ThrS; threonyl-tRNA synthetase	tRNA aminoacylation		-0.658 (1)	
NGO18231[f]	RpmD; 50S ribosomal protein L30	Ribosomal proteins: synthesis and modification		-0.592 (1)	
NGO0604	RpsA; 30S ribosomal protein S1	Ribosomal proteins: synthesis and modification			-0.930 (1)
NGO0442	50S ribosomal protein L25/general stress protein Ctc	Ribosomal proteins: synthesis and modification			-0.913 (1)
NGO0297	RpmI; 50S ribosomal protein L35	Ribosomal proteins: synthesis and modification			-0.885 (1)
NGO18311[g]	RplP; 50S ribosomal protein L16	Ribosomal proteins: synthesis and modification			-0.756 (1)
NGO1677	RpmA; 50S ribosomal protein L27	Ribosomal proteins: synthesis and modification			-0.619 (1)
**Purines, pyrimidines, nucleosides, and nucleotides**					
NGO0029	PyrE; orotate phosphoribosyltransferase	Pyrimidine ribonucleotide biosynthesis	-0.608 (1)		
NGO0441	ribose-phosphate pyrophosphokinase	Purine ribonucleotide biosynthesis	-0.602 (3)		
NGO0048	CarB; carbamoyl phosphate synthase large subunit	Pyrimidine ribonucleotide biosynthesis		-0.906 (1)	
**Regulatory functions**					
NGO0803[h]	putative GTP-binding protein	Other	-0.714 (1)		
**Transcription**					
NGO1785	Rne; ribonuclease E	RNA processing		-0.623 (1)	
NGO1856[i]	NusG; transcription antitermination protein NusG	Transcription factors			-0.763 (1)
**Transport and binding proteins**					
NGO2093	FetA; iron complex outermembrane recepter protein	Cations and iron carrying compounds	-2.447 (1)	-2.440 (2)	-2.008 (3)
NGO1935	EtfB; electron transfer flavoprotein beta subunit	Cations and iron carrying compounds	-0.954 (1)		
NGO0764	putative ABC-transporter, ATP-binding protein	Cations and iron carrying compounds	-0.766 (1)		
NGO1496[j]	TbpB; transferrin-binding protein B	Cations and iron carrying compounds		-2.418 (2)	
NGO1495	TbpA; transferrin-binding protein A	Cations and iron carrying compounds		-0.680 (1)	-1.623 (2)
NGO1378	ExbB; biopolymer transport protein ExbB	Unknown substrate			-0.951 (1)

[a] Functional role assignments (both main and JCVI sub-roles) for the *N. gonorrhoeae* proteins were downloaded from the JCVI-CMR website (http://cmr.jcvi.org/tigr-scripts/CMR/CmrHomePage.cgi).

[b] Average $\log_2$ values for biofilm/planktonic protein ratios. Values given in red meet the 2-fold cutoff threshold ($\log_2$ values $\leq$ -1.000).

[c] (N) represents the number of measurements (biological replicates) averaged.

[d] Proteins with more than one functional role assignment provided (alternate Main role/JCVI sub-role): NGO0346, Cellular processes/Chemotaxis and motility; NGO0639, Energy metabolism/Glycolysis/gluconeogenesis.

[e] NGO0108 tentatively reclassified based on orthology to *N. meningitidis* NMB1796 (99.4% identity), NMCC_0432 (98.8% identity), and NMC0426 (98.8% identity).

[f] NGO18231 tentatively reclassified based on orthology to *N. meningitidis* NMCC_1989 (98.4% identity), NMC0150 (98.4% identity), and NMB0160 (98.4% identity).

[g] NGO18311 tentatively reclassified based on orthology to *N. meningitidis* NMCC_1998 (100% identity), NMC0139 (100% identity), NMB0149 (100% identity), and NMA0122 (100% identity).

[h] NGO0803 tentatively reclassified based on orthology to *N. meningitidis* NMC1099 (99.7% identity) and NMCC_1078 (99.5% identity). Possible alternate Main role/JCVI sub-role assignments for this protein include: Cellular processes/Adaptations to atypical conditions (NMC1099 and NMCC_1078) and Protein synthesis/Translation factors (NMC1099 and NMCC_1078).

[i] NGO1856 tentatively reclassified based on orthology to *N. meningitidis* NMB0126 (100% identity) and NMCC_2018 (99.3% identity).

[j] NGO1496 tentatively reclassified based on orthology to *N. meningitidis* NMA2025 (72.3% identity), NMB0460 (62.2% identity), NMCC_1682 (57.4% identity) and NMC1691 (41.2% identity).
