# Supplementary material for: Proteomic Analysis of Neisseria gonorrhoeae Biofilms Shows Shift to Anaerobic Respiration and Changes in Nutrient Transport and Outermembrane Proteins
Source: PLoS One. 2012 Jun 6;7(6):e38303. doi: 10.1371/journal.pone.0038303 (PMC3368942; doi:10.1371/journal.pone.0038303)
Supplement: Table S7 — Average values (log2) for all of the quantified proteins detected in the study with p-values <0.05 and meeting a 1.5-fold cutoff threshold whose expression trends varied in different Extracts. ‘N’ indicates the number of measurements averaged. Functional classifications for the proteins were downloaded from the JCVI-CMR website (http://cmr.jcvi.org/tigr-scripts/CMR/CmrHomePage.cgi). (PDF) [file pone.0038303.s011.pdf]

Supplementary Table S7. Variable proteins in *N. gonorrhoeae* biofilms listed by functional roles[a]: Biofilm/Planktonic ratios expressed as log_2 values, 1.5-fold cutoff threshold (log_2 values either ≥ 0.585 or ≤ -0.585), p-values <0.05

Accession	Name	JCVI sub-role	Extract 1 Avg[b]	Extract 1 (N)[c]	Extract 2 Avg[b]	Extract 2 (N)[c]	Extract 3 Avg[b]	Extract 3 (N)[c]
**Amino acid biosynthesis**								
NGO1358	glutamate dehydrogenase	Glutamate family	0.888	(3)	-0.947	(2)	-1.726	(2)
**Cell envelope**								
NGO0332	putative reductase; dTDP-4-dehydrorhamnose reductase	Biosynthesis and degradation of surface polysaccharides and lipopolysaccharides	-0.807	(1)	0.856	(1)		
NGO1056	hypothetical protein; lipoprotein NlpD	Other			-1.151	(1)	0.954	(3)
**Cellular processes**								
NGO0328[e]	hypothetical protein; peroxiredoxin Q/BCP	Detoxification	-0.681	(1)	0.614	(1)		
NGO0926	putative peroxiredoxin family protein/glutaredoxin	Detoxification			0.610	(1)	-0.655	(1)
**Energy metabolism**								
NGO0617	Eno; phosphopyruvate hydratase; enolase	Glycolysis/gluconeogenesis	1.120	(3)	-0.900	(2)	-1.812	(1)
NGO0562	putative dihydrolipoamide dehydrogenase	Pyruvate dehydrogenase	0.996	(3)	-0.731	(3)	-1.142	(2)
NGO0915	dihydrolipoamide dehydrogenase	Pyruvate dehydrogenase	0.841	(3)	-0.967	(2)		
NGO2150	F0F1 ATP synthase subunit beta; F-type H+-transporting ATPase subunit beta	ATP-proton motive force interconversion	0.826	(1)	-1.543	(2)	-1.719	(1)
NGO0916	dihydrolipoamide succinyltransferase	TCA cycle	0.754	(1)			-2.314	(1)
NGO0217	ABC transporter, periplasmic binding protein, iron related; iron(III) transport system substrate-binding protein	Electron transport	0.618	(2)	-1.657	(3)	-0.958	(3)
NGO0200	phosphoenolpyruvate synthase	Glycolysis/gluconeogenesis	0.598	(3)	-1.351	(1)	-1.403	(1)
**Protein synthesis**								
NGO1853	RplJ; 50S ribosomal protein L10	Ribosomal proteins: synthesis and modification	0.648	(1)			-1.213	(1)
NGO2134	RpsU; 30S ribosomal protein S21	Ribosomal proteins: synthesis and modification	0.592	(1)			-0.852	(1)
NGO1825[f]	RplF; 50S ribosomal protein L6	Ribosomal proteins: synthesis and modification			-0.701	(1)	0.799	(2)
NGO1824[g]	RpsE; 30S ribosomal protein S5	Ribosomal proteins: synthesis and modification			-1.000	(1)	0.586	(1)
**Purines, pyrimidines, nucleosides, and nucleotides**								
NGO0799	inosine 5'-monophosphate dehydrogenase	Purine ribonucleotide biosynthesis	1.180	(3)	-0.716	(2)		
NGO0614	ribonucleotide-diphosphate reductase subunit alpha	2'-Deoxyribonucleotide metabolism	-0.656	(1)	1.086	(1)		
**Regulatory functions**								
NGO0460[d]	succinyldiaminopimelate transaminase; acetylornithine/N-succinyldiaminopimelate aminotransferase	Other	-0.622	(1)	0.681	(1)		
**Transcription**								
NGO0335	polynucleotide phosphorylase/polyadenylase; polyribonucleotide nucleotidyltransferase	Degradation of RNA	0.981	(3)	-1.093	(3)		
NGO1850	DNA-directed RNA polymerase subunit beta'	DNA-dependent RNA polymerase	0.805	(3)	-0.770	(1)	-0.595	(2)
NGO1851	RpoB; DNA-directed RNA polymerase subunit beta	DNA-dependent RNA polymerase	0.735	(3)	-1.045	(2)	-0.892	(2)
NGO1818	DNA-directed RNA polymerase subunit alpha	DNA-dependent RNA polymerase	0.585	(3)	-1.063	(3)		
**Transport and binding proteins**								
NGO1494	putative ABC transporter, periplasmic binding protein, polyamine	Amino acids, peptides and amines	1.287	(3)			-0.970	(1)
NGO1656[h]	hypothetical protein	Unknown substrate	0.760	(1)	-1.094	(1)		

[a] Functional role assignments (both main and JCVI sub-roles) for the *N. gonorrhoeae* proteins were downloaded from the JCVI-CMR website (http://cmr.jcvi.org/tigr-scripts/CMR/CmrHomePage.cgi).

[b] Average log_2 values for biofilm/planktonic protein ratios. Values given in red meet the 2-fold cutoff threshold (log_2 values either ≥ 1.000 or ≤ -1.000).

[c] (N) represents the number of measurements (biological replicates) averaged.

[d] Proteins with more than one functional role assignment provided (alternate Main role/JCVI sub-role): NGO0460, Unknown function/Enzymes of unknown specificity.

[e] NGO0328 tentatively reclassified based on orthology to *N. meningitidis* NMCC_0714 (97.9% identity).

[f] NGO1825 tentatively reclassified based on orthology to *N. meningitidis* NMCC_1992 (97.7% identity), NMC0147 (97.7% identity), and NMB0157 (97.7% identity).

[g] NGO1824 tentatively reclassified based on orthology to *N. meningitidis* NMB0159 (100% identity) and NMA0112 (100% identity).

[h] NGO1656 tentatively reclassified based on orthology to *N. meningitidis* NMCC_1797 (98.6% identity). Possible alternate Main role/JCVI sub-role assignments for this protein include: Transport and binding proteins/Amino acids, peptides and amines (NMCC_1797) and Cell envelope/Other (NMB0345, 98.6% identity).
